# High‐Contiguity Haplotype‐Resolved Genome Assembly of the Hexaploid *Actinidia valvata* Rootstock Sheds Light on Waterlogging Resistance Gene

**DOI:** 10.1111/pbi.70695

**Published:** 2026-05-29

**Authors:** Miaomiao Lin, Zhi Li, Cecilia H. Deng, Congcong Li, Zhengzhen Zhang, Yukuo Li, Qina Zhang, Peng Zhang, Leiming Sun, Mingyu Liu, Jinbao Fang, Xiujuan Qi

**Affiliations:** ^1^ National Key Laboratory for Germplasm Innovation & Utilization of Horticultural Crops Zhengzhou Fruit Research Institute, Chinese Academy of Agricultural Sciences Zhengzhou China; ^2^ Zhongyuan Research Center, Chinese Academy of Agricultural Sciences Xinxiang China; ^3^ Henan University of Urban Construction Pingdingshan China; ^4^ New Zealand Institute for Bioeconomy Science Auckland New Zealand

**Keywords:** *Actinidia valvata*, ERF, evolution, genomics, haplotype, kiwifruit, polyploid

## Abstract

Polyploidisation, creating redundant or diverged copies of the genome, is a major driving force in plant evolution, diversification and environmental adaptation, including for the kiwifruit genus (*Actinidia* Lindl.). We present a high‐contiguity, haplotype‐resolved genome assembly of the hexaploid *Actinidia valvata* rootstock cultivar ‘Zhongmikangzhen No. 2’ (ZK2), a novel rootstock and the first of its kind to receive Plant Variety Protection in China, exhibiting superior waterlogging tolerance. The assembly, ZK2, contains 174 chromosomes in 6 haplotypes (2*n* = 6*x* = 174), where > 82% of chromosomes per haplotype are telomere‐capped at one or both ends, including three haplotypes achieved with 100% telomeric representation. In total, 212 055 protein‐coding genes are predicted, or about 35 000 genes per haplotype on average. Our work on comparative genomics, including analyses of TE composition, collinearity, orthologs and phylogenies, strongly reveals an AABBBB subgenome structure derived from ancestral donors *A. polygama* (A1 and A2) and *A. macrosperma* (B1 to B4). Transcriptome analysis showed differential expressions between the homeologs under waterlogging stress, highlighting subgenome‐specific regulatory dynamics. A key example involves an ethylene‐response factor (*ERF*) gene: when the B1 copy of *ERF* was overexpressed in the kiwifruit transgenic lines, they illustrated an enhanced waterlogging resistance. In addition, this high‐quality haplotype‐resolved *A. valvata* genome assembly enables functional trait discovery, homeolog‐aware genome‐wide association studies, and targeted editing of beneficial alleles or homologous gene sets, supporting the breeding of resilient polyploid kiwifruit cultivars and rootstocks. This resource provides a foundation for future research in kiwifruit, particularly for rootstock‐mediated crop improvement.

## 1 Introduction

Subgenome dominance, wherein one subgenome exhibits asymmetric expression and functional contribution relative to others, has emerged as a central theme in understanding the evolutionary success of allopolyploids, which are widespread across the plant kingdom and have played a major role in speciation and adaptive innovation (Alger and Edger 2020; Schnable et al. 2011; Woodhouse et al. 2014). Each successful polyploidisation event leaves a mark within the genome that chronicles its history of hybridisation and genome duplication. For many modern polyploid species, however, particularly those shaped by multiple, reticulate hybridisation events such as species with hexaploidy or higher ploidy genomes, their precise origins remain enigmatic, thereby impeding efforts to unravel the molecular and evolutionary basis of subgenome dominance and their advantages to adaptation.

Kiwifruit (*Actinidia* Lindl.) is an important economic fruit worldwide, and its genome is characterised by extensive polyploidy, with ploidy levels ranging from diploids to decaplids and dodecaploids. Recurrent polyploidisation events have occurred across and within species, shaping the evolutionary diversification and complex traits of this genus (Faiz et al. 2026; Huang 2016). In our previous study, we selected the hexaploid *A. valvata* cultivar ‘Zhongmikangzhen No. 2’ (ZK2) as a kiwifruit rootstock, representing the first registered rootstock cultivar in China, with great potential due to its excellent resistance to waterlogging, a consequence of excessive rain and heavy soil; it also exhibits better compatibility with available scions and enhances their fruit size and flavour (Zhang et al. 2025). ZK2 will serve as a model material for studying the origins of polyploidy and the evolution of *Actinidia*, and will be instrumental in exploring resilience to abiotic stress and the interactions between scion and rootstock under waterlogging conditions. A high‐quality assembly of its genome will accelerate these studies.

Assembling the genome of polyploid plant species is challenging owing to the repeated polyploidisation events that have shaped them and the resulting sets of homoeologous and homologous chromosomes. In polyploid kiwifruit, this task is further complicated by hybrid lineages, interspecific introgression events, high heterozygosity, and the difficulty of distinguishing between homologous chromosomes (Liu et al. 2017). With the advancements of PacBio and high‐throughput chromosome conformation capture (Hi‐C) sequencing technologies, significant progress has been made in the successful assembly of polyploid genomes, such as with potato ( *Solanum tuberosum* ), alfalfa ( *Medicago sativa* ), sugarcane (*Saccharum* spp.), bamboo (*Bambusa* spp.), horseradish ( *Armoracia rusticana* ), chrysanthemum ( *Chrysanthemum morifolium* ) and cotton (*Gossypium* spp.) (Bao et al. 2023; Chang et al. 2024; Chen et al. 2020; Healey et al. 2024; Servant et al. 2015; Shen et al. 2023; Song et al. 2023; Wang et al. 2024; Zhang et al. 2018; Zheng et al. 2022). These achievements have provided valuable hints and knowledge applicable to the assembly of polyploid *Actinidia* genomes. Recently, chromosome‐level genome assemblies for several *Actinidia* species have been obtained, including diploid *A. chinensis* (Han et al. 2023; Yue et al. 2023, 2024), hexaploid *A. deliciosa* (Liu et al. 2024), diploid *A. arguta* (Akagi et al. 2023) and tetraploid *A. arguta* (Li, Song, et al. 2025; Lu et al. 2024; Zhang et al. 2024). These high‐quality assemblies offered insight into the evolution and polyploidisation‐driven adaptation of kiwifruit. Recently, the genome assemblies for tetraploid *A. valvata* and hexaploid *A. valvata* have been reported (Hu et al. 2025a; Zhang et al. 2026). However, given that the complex reticular evolution of kiwifruit genomes and gene introgression patterns have led to varying evolutionary histories among different species, and even within the same species, the assembly of high‐quality chromosome‐level genomes from different accessions is needed to fully understand the evolutionary origins of hexaploid and polyploidisation‐driven adaptation to various stress conditions.

In this study, we report a chromosome‐level assembly of the genome from the male hexaploid kiwifruit rootstock *A. valvata* ZK2. We employed PacBio Revio long read and Hi‐C sequencing to assemble the genome into six high‐quality chromosome‐level haplotypes, allowing the characterisation of the genome structure from each haplotype. Furthermore, we deciphered the history through which this hexaploid *A. valvata* was formed and identified its likely putative progenitors. We also identified a highly expressed homeologous copy of ERF that conferred greater tolerance to waterlogging when overexpressed. This assembly of the haplotype‐resolved *A. valvata* hexaploid genome will facilitate targeted editing of alleles or homeologous genes and potentially accelerate breeding of rootstock in kiwifruit.

## 2 Results

### 2.1 Genome Survey of the *A. valvata* Rootstock Cultivar ‘Zhongmikangzhen No. 2’

**FIGURE 1:**
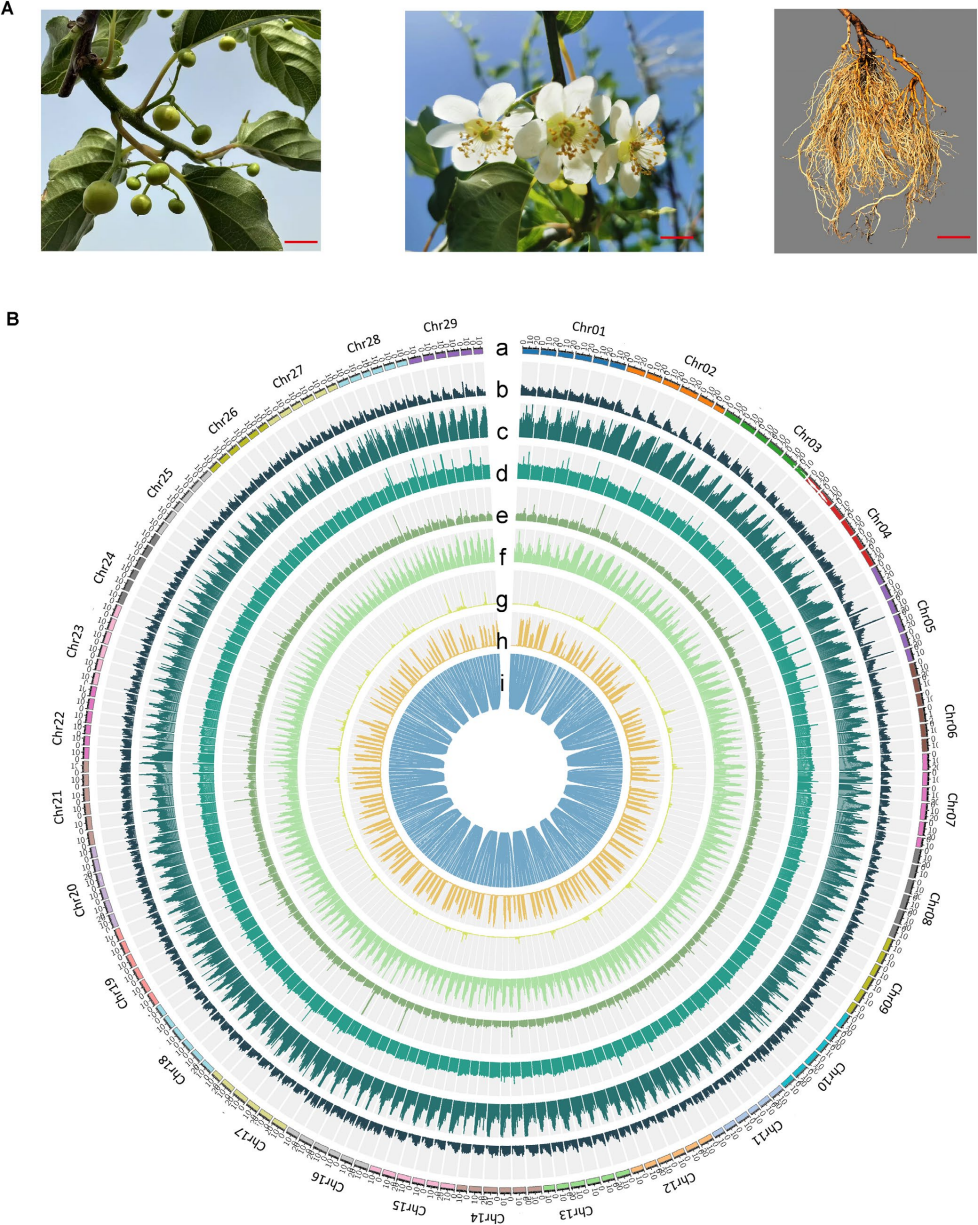
Genome assembly of hexaploid *A. valvata* ‘Zhongmikangzhen No. 2’ (ZK2). (A) Representative photographs of shoots, flowers and roots from ZK2 plants. The red bar represents 1 cm. (B) Circos plot summarising the features of the six ZK2 haps. From the outer to the innermost whorls: a, pseudochromosome; b, gene density; c, repeat elements; d, GC content; e, density of DNA transposable elements; f, density of long terminal repeat (LTR) transposons; g, insertion/deletion (InDel) density; h, single nucleotide polymorphism (SNP) density; i, intragenomic synteny. All densities and numbers were computed for 100‐kb sliding windows.

‘Zhongmikangzhen No. 2’ (ZK2) is a male rootstock cultivar of *A. valvata* (2*n* = 6*x* = 174). Its plants have flowers with 5–9 petals and yellow anthers, and a well‐developed root system (Figure 1A). We evaluated the size of the *A. valvata* genome by generating a total of 109.73 Gb of short paired‐end sequencing reads on the MGI platform, resulting in 102.12 Gb of clean sequencing reads. We estimated the heterozygosity in the *A. valvata* genome to be 1.5% according to a *k*‐mer analysis, and the estimated genome size is ~619 Mb (Figure S1; Table S1). The dominant class of AAAB (0.42), AB (0.22) and AAB (0.22) in the Smudgeplot reflects homeolog dosage asymmetry and unequal similarity among subgenomes, likely ancient allohexaploid with strong similarity as well as unequal divergence among the subgenomes (Figure S1).

### 2.2 Haplotype‐Resolved Genome Assembly of a Hexaploid *A. valvata* Rootstock Genome

**FIGURE 2:**
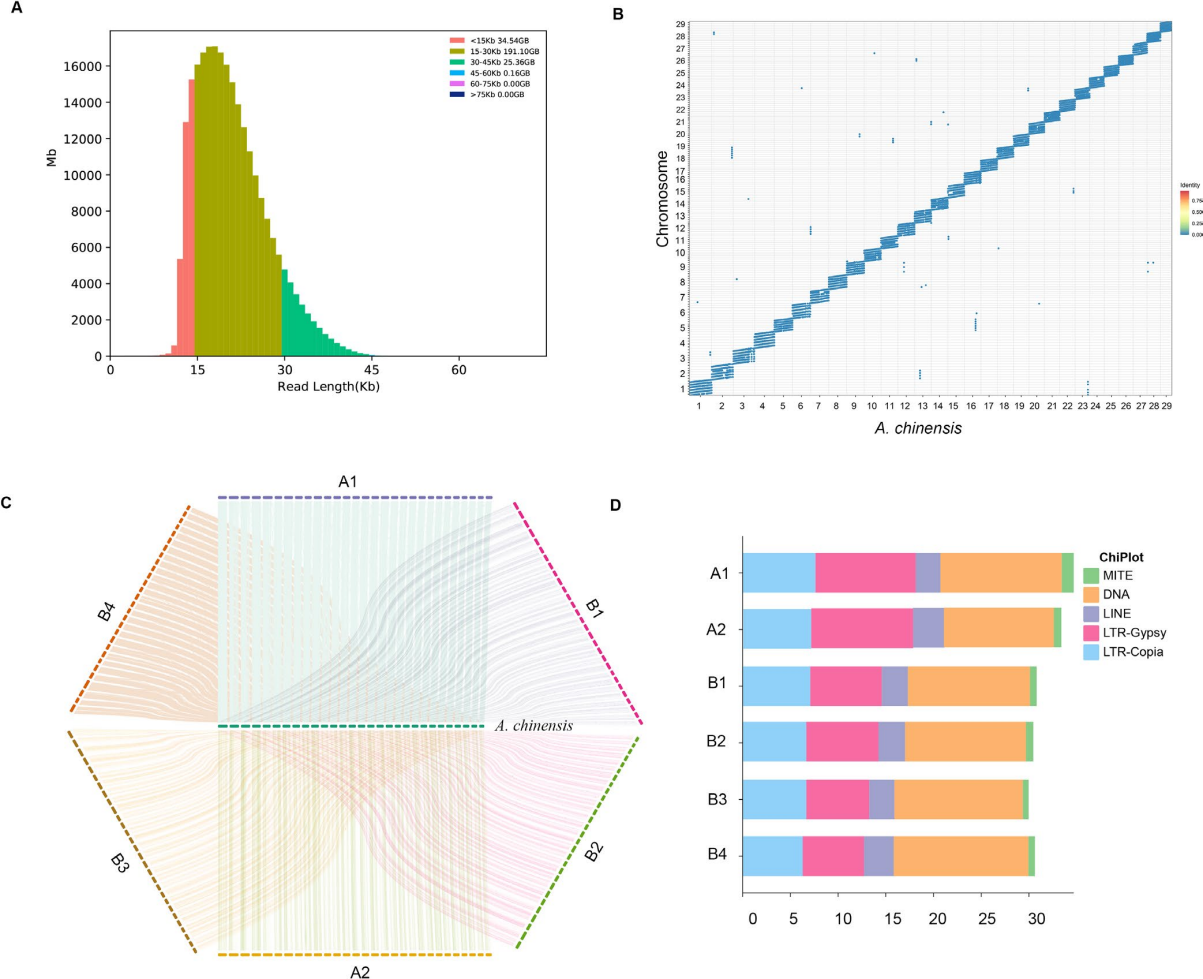
Intragenomic synteny of the *A. valvata* genome and annotation analysis. (A) Length distribution of HiFi sequencing. (B) Identity matrix across the haplotype‐resolved genome assembly showing synteny among the six chromosomes from each hap (A1, A2, B1, B2, B3 and B4) of *A. valvata* (y‐axis) and the chromosomes of *A. chinensis* (x‐axis). (C) Pairwise synteny plot between *A. chinensis* and each hap of *A. valvata* . Individual chromosomes are shown. (D) Number of different families of transposable elements across the six haps of *A. valvata* .

To obtain a high‐quality genome assembly, we generated 251.15 Gb of HiFi reads (~410 × coverage) using the PacBio Revio platform, with an N50 of 18 809 bp, a max length up to 68 187 bp, and a 99.5% ratio of reads with more than 10 kb length (Figure 2A; Figure S2; Table S2). In addition, we constructed and sequenced libraries for chromosome conformation capture sequencing (Hi‐C) on an MGI T7 platform, generating 572.6 Gb of clean reads (~950× coverage) (Table S3, Figure S2). Using these PacBio HiFi long reads and high Hi‐C data, we constructed a haplotype‐resolved high‐quality genome assembly for *A. valvata* , named ZK2 v1.0 (Figure 1B).

The *A. valvata* genome assembly consists of 174 chromosomes resolved into six haplotype‐resolved chromosome sets, designated hap A1, A2, B1, B2, B3, B4, based on synteny and phylogenetics analyses (Figures S3 and S4), as described in later sections. We oriented and assigned chromosome numbers for chromosomes in each hap according to their synteny to the 29 chromosomes in the *A. chinensis* ‘Hongyang’ v3 genome (Wu et al. 2019). The six resulting haps have a total length of 607.01, 604.99, 554.18, 518.57, 554.17 and 501.27 Mb, respectively (Table 1, Table S4). Interaction matrices based on Hi‐C data revealed strong signals along the diagonal for all chromosomes, indicative of local intra‐chromosomal interactions, demonstrating the high precision of the chromatin contact maps (Figure S5). A collinear analysis among the six haps of ZK2 and the ‘Hongyang’ v3 genome showed a high degree of global genomic synteny (Figure 2B,C).

**TABLE 1:** Summary statistics of the *A. valvata* genome assembly.

Genomic feature	A1	A2	B1	B2	B3	B4
Genome size (Mb)	607.01	604.99	554.18	518.57	554.17	501.27
Contig N50 (Mb)	20.83	21.02	19.04	18.26	18.76	17.05
Longest chromosome (Mb)	27.60	27.17	24.89	25.45	23.71	26.09
Shortest chromosome (Mb)	15.77	15.77	13.62	11.98	14.75	13.36
Genome BUSCO score (%)	98.70	98.60	96.10	91.70	96.30	92.30
GC content (%)	35.91	35.84	34.72	34.66	34.68	34.68
Total repeats (% of total)	50.04	49.51	44.80	45.05	44.94	43.51
TE size (% of total)	45.81	45.48	39.20	40.20	40.37	39.22
Number of genes	36 468	36 849	35 285	34 140	35 268	34 045
Gene BUSCO score (%)	95.04	95.97	93.43	92.94	94.30	92.57
Functionally annotated genes (%)	96.95	96.91	96.59	95.85	96.69	96.33
Number of haplotype‐specific genes	2175	2112	1485	1657	1446	1610

The quality of our genome assembly was comprehensively assessed using complementary approaches. Overall, we achieved 92.3% to 98.7% benchmarking universal single‐copy orthologs (BUSCO) completeness for the six haps (Table 1). The CRAQ analysis indicated high base‐level accuracy and overall reliability (Figure S6). Subphaser results further confirmed haplotype structure, clearly separating the 6 haplotypes into two distinct groups, which is consistent with phased subgenomes (AABBBB) (Figure S7). Merqury evaluation revealed that the six haplotypes achieved QV values ranging from 64 to 65, demonstrating high base‐level accuracy across the hexaploid assembly (Table S5). Telomeric repeat motifs were systematically assembled across all six haplotypes (Figure S8, Table S6), with > 82% of chromosomes per hap having telomere‐capping at one or both ends, including three haps (A1, A2, B3) that achieved 100% telomeric representation. The centromeres were detected by the CentroMiner module of quarTeT, and most of the centromeres were located near the middle parts of the chromosomes (Table S7). HiFi and Illumina data were mapped back to the assemblies to examine the coverage uniformity (Figure S9), sequencing depth distribution (~69× to 77× on average across the six haps), as well as quantifying collapsed errors (1.3% to 5.3% or 2.6% to 3.7% across the haps using HiFi or Illumina data respectively) (Table S8). In addition, super‐long ONT data analysis revealed a switch error rate of 6.28% (Figure S10).

### 2.3 Gene and Repeat Annotation Across Haplotype‐Resolved Assemblies

Our structural annotation revealed that transposable elements (TEs) accounted for 39.2%–45.8% of the entire DNA space across the six haps. Specifically, A1 and A2 had higher TE contents (about 45%) compared with hap Bs (~40%) (Table 1). Long terminal repeats (LTRs) were the most common type of TE, reaching 20.8%–29.4% across the six haps, while DNA‐type TEs represented 12.4%–14.1% of the *A. valvata* genome (Figure 2D, Table S9). The pattern of TE landscape was quite similar among the 6 haps (Figure S11), with a major peak at ~20% divergence, representing a TE amplification burst in the past, while A2 showed a second peak at around 2% divergence for a recent burst, and B4 exhibiting a distinct peak at 28% representing an earlier bust of TE activity (Figure S11).

Integrating transcriptome alignments, homology‐based searches, and *de novo* predictions on the soft‐masked genome, we predicted 212 055 protein‐coding genes across all 174 chromosomes. About 95.85%–96.95% of the predicted genes were successfully assigned potential functions; among them, 78.5%–80.4% with Swissprot annotation, 39.3%–40.6% with the Kyoto Encyclopedia of Genes and Genomes (KEGG), 52.9%–54.5% with the EuKaryotic Orthologous Groups (KOG), 60.6%–62.45% with Gene Ontology (GO) and 95.8%–96.9% with the non‐redundant NR database at NCBI (Table S10). Furthermore, we identified 4110 transfer RNA (tRNA), 10 702 ribosomal RNA (rRNA) and 8485 non‐coding RNA (ncRNA) loci (Table S11). A BUSCO analysis showed that 92.6%–96.0% of conserved plant genes are present in our annotation (Table 1, Table S12).

We conducted a homeologs‐aware gene annotation across the haplotype‐resolved allohexaploid assembly. Highly similar gene copies located at syntenic chromosomal loci across all the six haps (homologous or homoeologous) were grouped as a homoeologous gene set, representing copies derived from the two ancestral subgenomes (AABBBB). In this study, the term ‘homoeologous’ is used to describe corresponding gene copies originating from distinct ancestral subgenomes in the hexaploid genome. This usage only reflects subgenomic origin rather than segregation behaviour or inheritance patterns, which were not investigated here. Within each homoeologous gene set, individual gene copies correspond to homeologs rather than allelic variants. We thus identified a total of 137 523 homoeologous gene sets with at least two gene copies across the *A. valvata* genome. Among these, 76 068 gene sets have six homoeologous copies, 33 030 genes have five copies, 11 428 genes have four copies, 7599 genes have three copies and 9398 genes have two copies (Table S13). The frequency of gene sets with six homoeologous copies is dominant, with many of them also accompanied by further paralogous copies within the same haplotype‐resolved assembly.

### 2.4 Structural Variations and Haplotype‐Level Genome Comparisons

**FIGURE 3:**
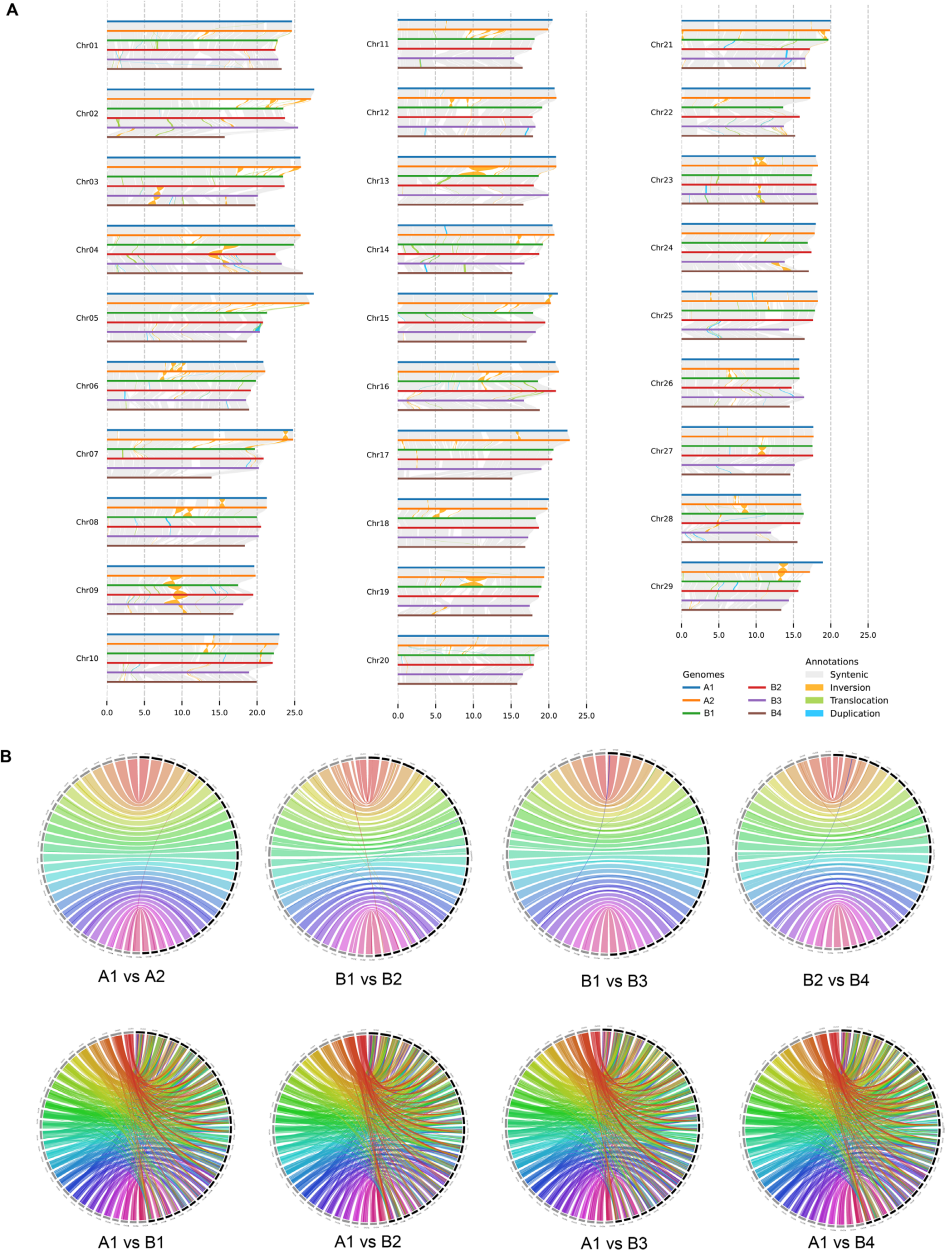
Comparison of the different haplotype‐resolved genome assemblies of the *A. valvata* genome. (A) Extent of the structural diversity of the haplotype‐resolved pseudochromosomes from *A. valvata*. All 174 chromosomes (29 chromosomes per haplotype) are drawn to scale. Synteny between individual haplotype‐resolved pseudochromosomes is shown as grey lines, inversions as orange lines, translocations as green lines, and duplications as blue lines. (B) Pairwise synteny analysis between the indicated pair of haplotype‐resolved genome assemblies: A1 versus A2; B1 versus B2; B1 versus B3; B2 versus B4; A1 versus B1; A1 versus B2; A1 versus B3; A1 versus B4.

We performed a pairwise comparative analysis between the six haplotype genomes, which revealed that the *A. valvata* genome is organised as two groups of chromosomes, each sharing many genomic structural features. For instance, A1 and A2 show similar genomic features, including comparable assembly sizes, similar repeat contents and similar numbers of genes (Table 1), while the other four haps (B1, B2, B3, B4) have similar genomic structural features. We also detected large inversions between the two groups (Figure 3A). We compared the two groups to identify all polymorphisms across the *A. valvata* genome, and identified 1 189 928, 2 254 746, 2 068 864, 1 156 964, 2 344 501 and 1 942 204 single nucleotide polymorphisms (SNPs), 93 557, 92 107, 123 888, 112 694, 130 916 and 102 104 insertions; 95 919, 93 187, 115 615, 105 531, 122 616 and 94 912 deletions; 273, 307, 1105, 1378, 1232 and 1204 translocations; 407, 560, 2375, 2901, 2429 and 2918 duplications; 808, 570, 4154, 4649, 4184 and 4789 inversions in A1, A2, B1, B2, B3, B4, respectively (Figures 1B and 3A). In particular, we detected a high density of DNA‐TEs on chromosomes 2 and 16 in B1, Chr 16 and 27 in B4, a large amount of InDels on chromosomes 3 in A1, Chr 10 and 28 in B2, and a high density of SNPs in all assemblies (Figure 1B, Tables S9 and S14).

For each chromosome, the structure variation between the six copies was visualised in Figure 3A. Strong intra‐group collinearity was observed among group A (A1–A2) and B (B1–B4) assemblies; in contrast, inter‐group comparison showed a lower scale of synteny (Figure 3B), suggesting that subgenomes A and B might be derived from distinct ancestral donors.

### 2.5 Phylogenetics Analysis of the Hexaploidisation That Led to Haplotype‐Resolved *A. valvata* Genome Through Genome‐Wide Orthologous Study

We used two algorithms, orthoMCL (Li et al. 2003) and orthoFinder (Emms and Kelly 2019) to identify orthologous gene sets, infer gene trees and species tree, across seven kiwifruit species, namely hexaploid *A. chinensis var*. *A. deliciosa* , tetraploid *A. arguta* , diploid *A. eriantha* , diploid *A. chinensis* , diploid *A. polygama* , diploid *A. hemsleyana* and tetraploid *A. macrosperma* , and four other plant species *Rhododendron delavavi*, *Vitis vinifera* , *Arabidopsis thaliana* and *Oryza sativa* . OrthoFinder created hierarchical orthogroups (HOGs) and generated the species phylogenetic tree (Figure S12), clearly illustrating that group A (A1–A2) and B (B1–B4) were closely clustered to two different species, *A. polygama* and *A. macrosperma* , respectively. Combining all the observations from syntenic checks (Figures S3 and S4), orthologous analysis and phylogenomic evaluation, we proposed that the two groups for *A. valvata* genome originated from different progenitors: *A. macrosperma* and *A. polygama* , with *A. macrosperma* being one of the parents, while *A. polygama* was likely a sister of the second parent.

**FIGURE 4:**
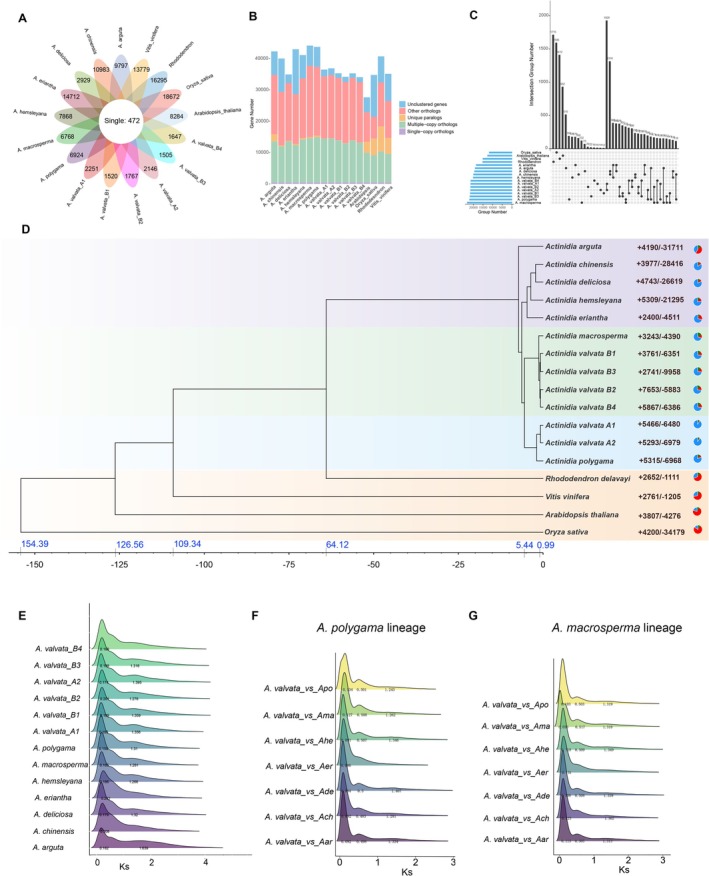
Phylogenetic relationships, comparative genomics, and evolutionary analysis of the *A. valvata* genome. (A) Venn diagram showing the number of gene families in the *A. valvata* genome, the genomes of other *Actinidia* species, and other plant species. (B, C) Number (B) and UpSet plot (C) of gene families unique to each plant species or shared with other plant species. (D) Phylogenetic tree showing the divergence times of each species, as well as the number of expanded and contracted gene families. The pie chart on the right represents gene expansion and contraction. (E) *Ks* analysis of paralogous gene pairs in kiwifruit species. (F, G) *Ks* analysis of orthologous gene pairs between the genes assigned to the haplotype‐resolved assemblies originating from subgenome A versus *A. polygama* (F) or subgenome B versus *A. macrosperma* (G) and other kiwifruit species.

For the orthoMCL result, we identified 472 shared single‐copy genes shared by *A. valvata* and all other plant species (Figure 4A). We also identified 2251, 1520, 1767, 2146, 1505 and 1647 unique or unclustered genes in each of the six haplotype‐resolved genome assemblies of *A. valvata* (Figure 4A–C). Using the above single‐copy genes, we then re‐constructed the phylogeny using the subgenome‐specific single‐copy orthologous genes; in consistent with the orthoFinder result, the six *A. valvata* haps clustered into two different groups, with A1 and A2 assemblies grouped with *A. polygama* , while B1 to B4 assemblies clustered closer to *A. macrosperma* in a separate branch. An estimation of divergence times indicates that the major lineages *A. macrosperma*, *A. valvata* and *A. polygama* diverged approximately 5.44 million years ago (Mya), subsequent branching of *A. macrosperma* and *A. valvata* subgenome group B (B1 to B4) occurred around 1.33 Mya, whereas diversification of *A. polygama* and *A. valvata* group A (A1, A2) happened earlier at approximately 1.92 Mya (Figure 4D).

We detected evidence for three recent whole‐genome duplication (WGD) events at Ad‐α, Ad‐β and Ad‐*γ* in the *A. valvata* genome assembly, results that are largely consistent with previous analyses of genomes from other kiwifruit species (Liu et al. 2024; Zhang et al. 2024) (Figure 4E). A *Ks* distribution analysis among the two *A. valvata* subgenomes and the genomes of other kiwifruit species revealed clear divergence peaks, with major *Ks* values ranging from 0.04 to 0.19, and a shared ancient peak around *Ks* 1.2–1.4, suggesting a common ancestral WGD event (Figure 4F,G). We calculated the lowest *Ks* value for the *A. valvata* A1 assembly and *A. hemsleyana*, indicating a relatively weaker sequence divergence, while *A. macrosperma* and *A. polygama* showed higher *Ks* peaks, suggesting an earlier divergence time. Notably, the results of this *Ks*‐based analysis differed from those derived from the phylogenetic tree, where *A. hemsleyana* appears more distantly related to *A. valvata* (Figure 4F). Such discrepancies likely arose because *Ks* distributions can be influenced by gene duplication, differential substitution rates, or subgenome‐specific histories in polyploid genomes, whereas single‐copy phylogenies primarily reflect species‐level divergence. Therefore, *Ks*‐based divergence patterns should be interpreted as reflecting the evolutionary history of the two *A. valvata* subgenome compartments rather than direct measures of relatedness.

**FIGURE 5:**
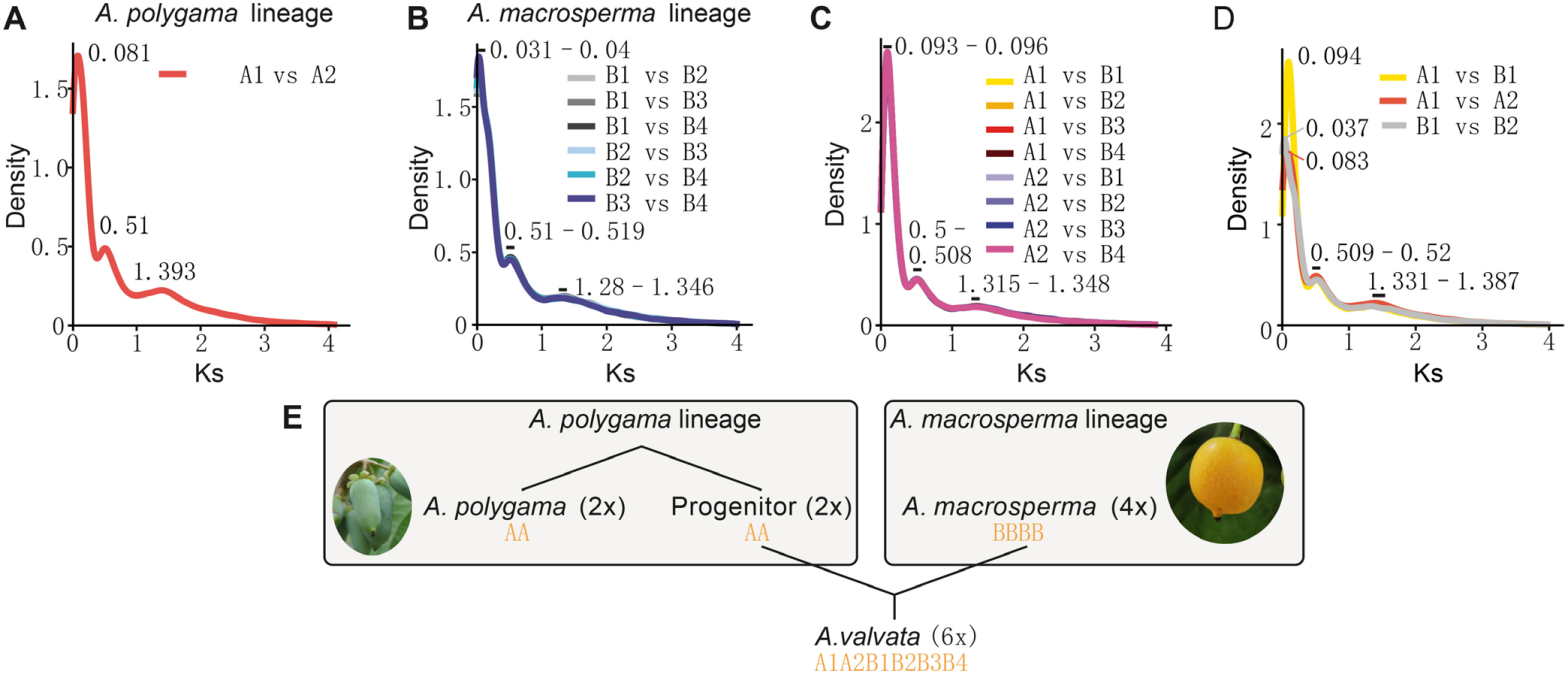
Whole‐genome duplication analysis of the two subgenomes of *A. valvata* . (A) *Ks* comparison between A1 and A2, which belong to the *A. polygama* lineage. (B) *Ks* comparison among B1, B2, B3, B4, which belong to the *A. macrosperma* lineage. (C, D) *Ks* comparison of the *A. polygama* lineage and *A. macrosperma* lineage. (E) Illustration of the evolution of *A. valvata* ZK2.

Further, to confirm that the two subgenomes defined by the two branches of the phylogenetic tree originated from different ancestors, we calculated the *Ks* values between the *A. polygama* ‐derived subgenome, the *A. macrosperma‐*derived subgenome and between these two subgenomes (Figure 5A–D). The *Ks* value for the *A. polygama* branch was 0.08, that of the *A. macrosperma* branch was 0.03, and the *Ks* value between these two branches was 0.09, indicating that the *A. polygama* and *A. macrosperma* branches originated from different ancestors, and that the differentiation times between the two subgenomes are different (Figure 5E).

### 2.6 Haplotype‐Level Analysis of the *ERF* Gene Family in a Hexaploid Rootstock of *A. valvata*

The genomic structure analysis and evolutionary analysis above indicate that the *A. valvata* genome can be divided into two subgenomes: group A originating from *A. polygama* and B from *A. macrosperma* . Gene family dynamics also differed noticeably between the two lineages (pie charts in Figure 4D): in the A subgenomes, gene expansion (2652 and 2761 in A1 and A2) is doubled to the contractions (~1100 to ~1200 in A1 and A2); while group B exhibits substantially higher gene expansion (5293 to 5867 in the four Bs) and contraction (6386 to 6979) comparing to group A (Figure 4D). GO and KEGG analysis revealed that the expanded gene families are significantly enriched in pathways related to ‘cellular respiration regulation’, ‘oxidoreductase activity’, ‘starch and sucrose metabolism’ and ‘MAPK signaling pathway–plant’. These pathways are closely associated with hypoxia and flooding tolerance, reflecting enhanced capacity for redox balance, energy metabolism adjustment, and stress signal transduction. These pathways are also under the control of the ethylene signalling pathway. Given the *ERF* gene family's central role in the ethylene signalling pathway (Chen et al. 2024), we chose it as a representative example to demonstrate the power of utilising a haplotype‐resolved genome for haplotype‐specific discovery and functional validation (Table S15, Figure S13).

**FIGURE 6:**
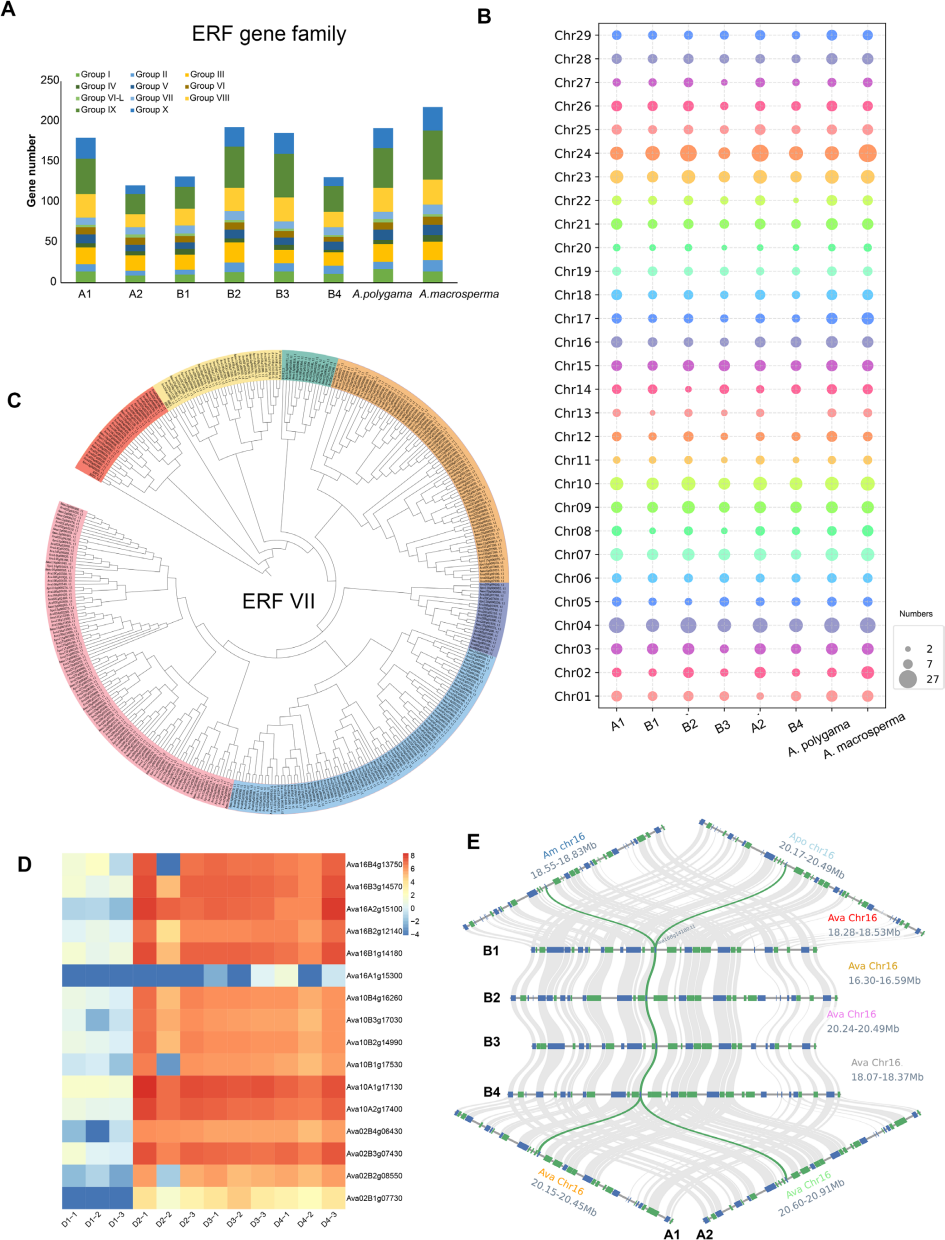
Genome‐wide identification of the *ERF* gene family in *A. valvata* , *A. polygama* and *A. macrosperma* . (A) Number and type of *ERF* in the assemblies. Each bar represents one hap or assembly, with colours indicating *ERF* group type, and height for number of genes. (B) Number of *ERF* genes on each chromosome. (C) Phylogenetic tree of *ERF* clade VII genes in the *A. valvata* genome. (D) Heatmap of expression levels for the six haplotypes of the *ERF* gene 16B1g14180 and its orthologous genes. D1, D2, D3, D4 represent root for ZK2 under waterlogging treatment for 0, 12, 24 and 72, respectively. (E) Local collinearity plot showing the synteny of the *ERF* gene 16B1g14180 across the six haplotype‐resolved *A. valvata* genome assemblies, *A. polygama* and *A. macrosperma* . The same chromosome fragment is shown, with 16B1g14180 and its homoeologous or homologous genes connected by green lines. Gene models are shown as boxes alternating between blue and green.

In our previous study, we showed that the waterlogging tolerance of hexaploidy *A. valvata* was superior to *A. polygama* and *A. macrosperma* , although the latter has excellent waterlogging resistance (Bai et al. 2019). To reveal the genomic basis of this superior trait, we analysed the *ERF* gene family in ZK2 assembly and the two putative progenitors. In each hap, we detected a decreased amount of *ERF* gene families than in its progenitors (Figure 6A,B). However, the type of ERF was consistent, as our previous study showed that *ERF*‐VII family members strongly respond to waterlogging (Li et al. 2022). We reconstructed a phylogenetic tree for the *ERF*‐VII family (Figure 6C), and measured the expression levels of these genes in *A. valvata* under waterlogging stress (Table S16). Evidence for subgenome dominance emerged in the hexaploid *A. valvata*, through an *ERF* gene with six homeologs (16A1g15300, 16B1g14180, 16B2g12140, 16A2g15100, 16B3g13750, 16B4g14570). Genes belonging to the same HOG likely have originated from a single ancestral gene and may retain similar functions. Under waterlogging treatment, the expression pattern of the six homeologs of this *ERF* gene and additional copies in the same HOG (Figure 6D) illustrated that only one copy, Ava16A1g15300, failed to respond to waterlogging. The other five of the homeologs were highly expressed, suggesting that the tolerance of *A. valvata* to this stress is linked to gene dosage effects. We conducted a collinearity analysis and found that this gene also exists in the putative ancestors *A. valvata* and *A. macrosperma* (Figure 6E).

### 2.7 Functional Characterisation of a Candidate *ERF* Gene by Transgenic in Kiwifruit

**FIGURE 7:**
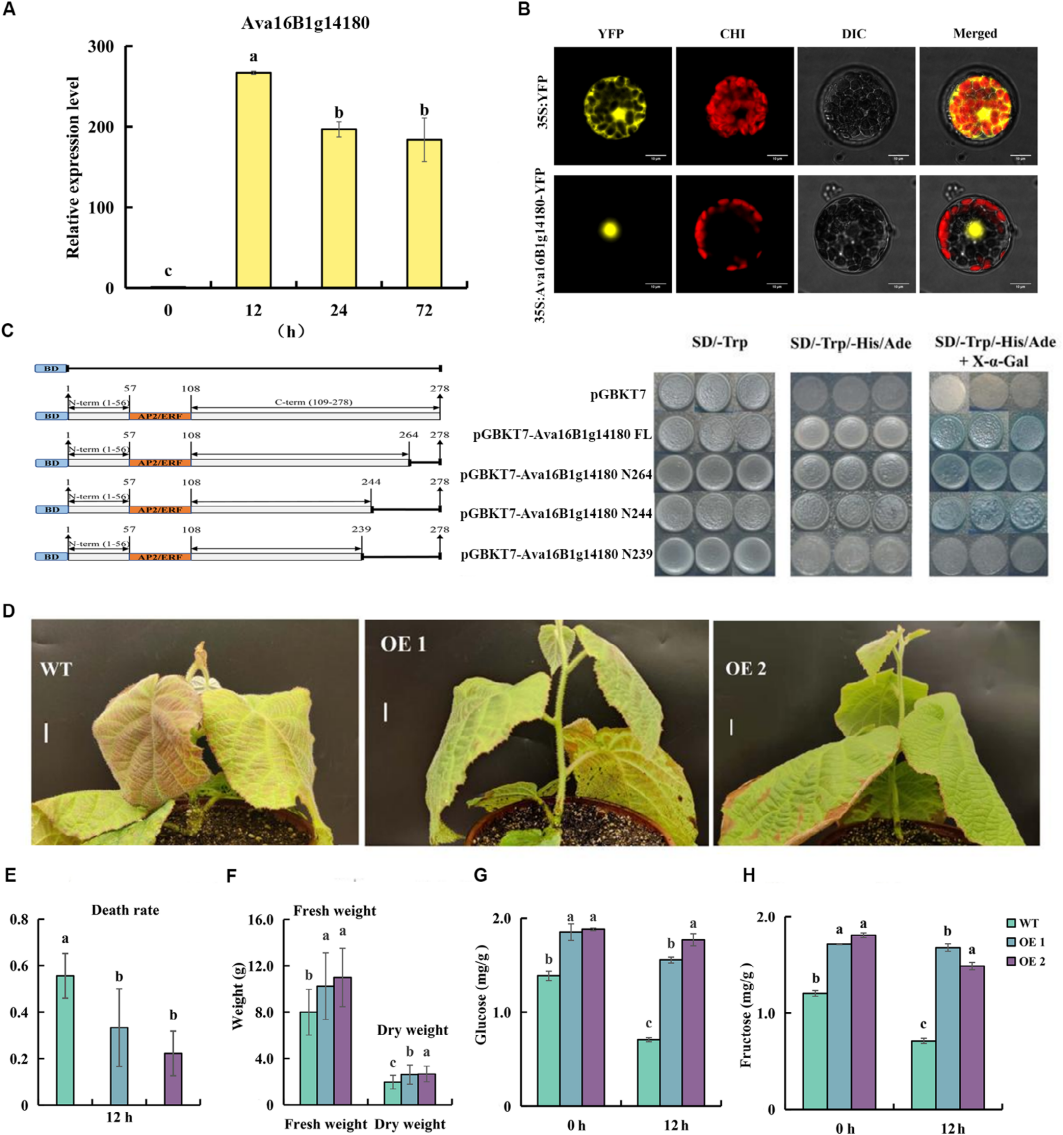
Identification and characterisation of the waterlogging‐resistance *ERF* gene 16B1g14180 in kiwifruit. (A) Relative expression levels of 16B1g14180 in the roots of *A. valvata* plants after 0, 12, 24, or 72 h of waterlogging stress. (B) Subcellular localisation of the protein encoded by 16B1g14180. The indicated plasmids were transfected into Arabidopsis protoplasts, and YFP signals were visualised using a confocal laser scanning microscope. YFP, yellow fluorescent protein; Chl, chlorophyll autofluorescence; DIC, differential interference contrast. (C) Transactivation activity assay of the protein encoded by 16B1g14180. Left, diagrams of the pGBKT7 fusion constructs used in the assay. Right, representative photographs of yeast colonies harbouring the indicated plasmids spotted on synthetic defined (SD)/−Trp medium, SD/−Trp/−His medium, or SD/−Trp/−His medium +X‐α‐Gal. (D) Representative photographs of wild‐type (WT) kiwifruit plants and kiwifruit plants overexpressing 16B1g14180 after 14 days of waterlogging stress. OE, overexpression. (E) Death rate of WT and OE plants under 12 h waterlogging treatment. (F) Fresh weight and dry weight of leaves of WT and OE plants under 12 h waterlogging treatment. (G, H) The content of glucose and fructose of roots in WT an OE under 12 h of waterlogging stress.

The two allelic *ERF* gene pairs 16A1g15300 versus 16A2g15100 and 16B1g14180 versus 16B3g14570 are 100% identical at the cDNA sequence level, whereas 16B2g12140 lacks a portion of the coding sequence (Figure S14). We chose 16B1g14180 from the *A. macrosperma* group with a higher expression level in response to waterlogging to test its potential. 16B1g14180 expression was highly induced in response to waterlogging (Figure 7A, Table S17). We examined the subcellular localisation of its encoded protein as a fusion to the yellow fluorescent protein (YFP); following transfection of protoplasts prepared from Arabidopsis plants with the encoding plasmid, we observed yellow fluorescence in the nucleus, indicating that this protein is a nuclear protein (Figure 7B). The protein encoded by 16B1g14180 showed clear transactivation activity when produced in yeast ( *Saccharomyces cerevisiae* ) cells, and sequential deletions revealed that the N‐terminal segment (amino acids 239–244) is critical for reporter gene activation (Figure 7C).

We generated transgenic *A. chinensis* kiwifruit seedlings overexpressing 16B1g14180; after 14 days of waterlogging stress imposed on potted seedlings, the wilting degree of the leaves and terminal buds of the overexpressing plants was significantly lower than that of the wild type, which was accompanied by a significantly lower death rate in the transgenic plants relative to the wild type, along with greater aboveground fresh and dry weights (Figure 7D–F). Root glucose and fructose contents were significantly lower in wild‐type plants than in the overexpression lines, regardless of growth conditions (*p* < 0.05). Following 12 h of waterlogging, the wild type showed a marked drop in glucose and fructose contents; in particular, glucose levels were 49.0% lower in wild type, but only 16.0% lower in 16B1g14180‐OE1 and 5.9% lower in 16B1g14180‐OE2 seedlings (Figure 7G,H). These results indicate that 16B1g14180 enhances the waterlogging tolerance of transgenic kiwifruit plants.

## 3 Discussion

Subgenome dominance, wherein one subgenome exhibits asymmetric expression and functional contribution, has emerged as a key theme in understanding polyploid evolution. Polyploidy is very common across the plant kingdom and has played a central role in shaping plant evolution, species diversification and trait innovation. Many horticultural crops are polyploid, and well‐characterised polyploid genomes have greatly advanced our understanding of their evolutionary origins and adaptive advantages. Several polyploid kiwifruit genomes have been published (Hu et al. 2025b; Li, Kuhl, et al. 2025; Li, Song, et al. 2025; Lu et al. 2024; Zhang et al. 2024). Our work addresses a gap in hexaploidy *A. valvata* genomics by providing a high contiguous, well annotated assembly, focusing on a novel cultivar, ‘Zhongmikangzhen No. 2’ (ZK2), the first kiwifruit rootstock received Plant Variety Rights protection in China. Compared with a recent published genome on a wild germplasm (Zhang et al. 2026) containing 500 to 700 gaps across the six subgenomes (four of which is smaller than 500 MB in assembled genome size), our assembly demonstrates superior contiguity, with two subgenomes achieved < 20 gaps, and the worst one with about 300 gaps. Beyond the gaps, the size of our subgenomes (501 Mb to 607 Mb) shows uniformity and better aligns with the known *Actinidia* genome size. We present a high‐contiguity with less gaps, haplotype‐resolved, more complete genome assembly with improved telomere caps (11 to 26 T‐to‐T chromosomes in the six subgenomes, comparing to the Zhang et al. (2026) with 3 to 9 chromosomes with telomeres at both ends) of the kiwifruit rootstock variety ZK2. This work increases haplotype resolution, reduces gaps and provides a robust foundation for polyploid *A. valvata* genomics study, enabling reliable homoeologous haplotype‐specific genomic studies related to complex trait loci. Given the widespread use of *A. valvata* ZK2 as a waterlogging‐tolerant rootstock, this genome resource offers an important foundation for functional studies and rootstock‐focused breeding applications.

The origins of polyploid kiwifruit species are complex owing to the diverse geographical conditions and introgressive hybridisation. Moreover, kiwifruit plants have a relatively short cultivation history, most kiwifruit species persist in natural wild populations, and parental information is lacking (Xu et al. 2026). The same situation applies to hexaploid species *A. valvata* as well. Previous molecular marker analysis had suggested a close relationship between *A. valvata* and *A. tetramera* (Chat et al. 1996). In our study, the genomic structural features of the six haps generated in this study help shed light on the origins of hexaploid *A. valvata* . We concluded that ZK2 contains two distinct subgenomes, each derived from a different progenitor species; that is consistent with the genome size based on the *k*‐mer results. We therefore propose that *A. macrosperma* represents the progenitor of this first subgenome lineage, whereas the A1 and A2 assemblies cluster with *A. polygama* and diverged from it approximately 1.92 Mya (Figure 4D), and that *A. polygama* is the sister lineage to one of the parents contributors of this second subgenome. Furthermore, analyses of expanded or contracted gene families support the conclusion that hexaploid *A. valvata* comprises two distinct subgenomes.

Whole‐genome duplication (WGD) serves as a potent evolutionary driver for genome expansion and trait formation (Gao et al. 2025). The genomes of most eudicot species, including kiwifruit, have undergone at least one ancient WGD event (*Ad‐γ*) as well as two more recent lineage‐specific WGDs (*Ad‐α* and *Ad‐β*) (Aköz and Nordborg 2019; Shi et al. 2010; Zhang et al. 2024). Our *Ks* analysis revealed evidence for three WGD events in *A. valvata* as well. Given that the six haplotype genomes belong to two lineages that have undergone different WGD events and constitute two subgenomes (Figure S5). We conclude that hexaploid *A. valvata* is an allopolyploid species formed through hybridisation between *A. macrosperma* and a sister species of *A. polygama* (Figure 5E). Collectively, the results of *Ks* and phylogenetic analyses indicated that the polyploid genome of *A. valvata* may comprise two subgenomes of divergent ancestral origins, and that a block in chromosome separation during gamete formation may have contributed to the emergence of this species.

The evolution of polyploid kiwifruit shares many similarities. For instance, *A. deliciosa* and *A. valvata* both originated from the hybridisation of two species; however, whereas 13 out of 39 chromosomes in *A. deliciosa* exhibited structural rearrangements (Li, Kuhl, et al. 2025), a much higher proportion of chromosomal rearrangement occurred in *A. valvata* . These may lead to multiple differences in traits of the kiwifruit. Gene expression dominance is frequently associated with differential transposable elements (TE) load between subgenomes (Huang et al. 2020; Ramakrishnan et al. 2022; Woodhouse et al. 2014). We also find this evidence in hexaploid kiwifruit. In tetraploid *A. valvata* , higher gene expression levels in the dominant subgenome were associated with lower TE content (Hu et al. 2025a), consistent with previous observations that TEs repress gene expression via epigenetic silencing (Woodhouse et al. 2014). In our study, hexaploid remained the higher expression in the lower TE content subgenome.

Beyond TE proportion, WGD events and subsequent gene expression dosage effects may further contribute to enhance gene expression in hexaploid ZK2. The superior tolerance to waterlogging observed in ZK2 likely stems from the selective retention of a large ancestral repertoire of tolerance‐associated genes and a complex regulatory network, rather than from the recent acquisition of novel genes via hybridisation. The *ERF* gene family analysis indicated that the formation of species of *A. valvata* ZK2 retained and expanded the *ERF* family, which is related to the waterlogging trait. In this study, we analysed the *ERF* gene family across the haplotype genomes and their putative progenitor species, identifying a higher number of *ERF* genes compared to the progenitor genomes. Concurrently, the gene dosage effect resulting from the merger of two genomes in the initial hybrid, combined with subgenome‐specific expression dominance, may have enhanced the expression levels of specific genes and thereby improved stress tolerance (Paterson et al. 2012; Woodhouse et al. 2010). We specifically examined a waterlogging responsive *ERF* gene harbouring six homeologous genes across the haplotype genomes; unlike other published *ERF* genes (Bai et al. 2021), only the A1 allele (derived from the *A. polygama* lineage) of the *ERF* gene had a low response to waterlogging, whereas the expression levels of the other alleles of this gene were significantly elevated. Further, overexpression of the *ERF* gene (Ava16B1g14180) conferred enhanced tolerance to waterlogging in transgenic kiwifruit plants. We conclude that hybridisation through interspecific crossing and increased higher gene dosage may contribute to the enhanced waterlogging resistance observed in hexaploid *A. valvata* , providing a clear example of subgenome dominance contributing to stress adaptation in polyploid kiwifruit advantage and shedding light on its evolutionary trajectory (Van de Peer et al. 2017).

## 4 Conclusion

Overall, we have generated a high‐contiguity haplotype‐resolved genome assembly for hexaploid *A. valvata* novel rootstock cultivar ZK2, which serves as a reference resource for targeting editing of advantageous homeologs or alleles, and accelerate precise breeding of kiwifruit rootstock cultivars.

## 5 Materials and Methods

### 5.1 Plant Materials, Library Preparation and DNA Sequencing

The *A. valvata* cultivar ZK2 was planted at the Zhengzhou Fruit Research Institute, Chinese Academy of Agricultural Sciences (CAAS), China (113° E, 34° N). Young leaves were collected from one‐year‐old shoots, frozen in liquid nitrogen, and stored at −80°C for short‐read (MGI), PacBio HiFi, and Hi‐C sequencing. Genomic DNA was isolated by the CTAB method. DNA concentration was determined with a Qubit Fluorometer, DNA integrity and purity were assessed by agarose gel electrophoresis. For MGI sequencing, libraries were constructed with an MGIEasy Universal DNA Library Prep Kit V1.0 following the standard protocol, the single‐stranded circle DNA (ssCir DNA) was formatted as the final library and qualified libraries were sequenced on an MGISEQ 2000 instrument. For PacBio HiFi sequencing, SMRTbell libraries in the target fragment size were constructed for sequencing according to the standard protocol (Pacific Biosciences, CA, USA) using either 10 kb or 20 kb preparation solutions. The SMRTbell library was then purified using AMPure PB beads and checked on an Agilent 2100 Bioanalyzer (Agilent Technologies, USA) to assess the size distribution of library fragments. Sequencing was performed on a PacBio Revio instrument. For Hi‐C sequencing, genomic DNA was isolated, ligated and sheared into 300–600 bp fragments, before being blunt‐end repaired and A‐tailed, followed by purification through biotin‐streptavidin‐mediated pull down. Finally, the Hi‐C libraries were quantified and sequenced on an MGI‐2000 platform. For ONT Ultra‐long sequencing, genomic DNA was extracted and purity, the DNA library was prepared by the Ligation sequencing 1D Kit (SQK‐LSK114, Oxford Nanopore Technologies, Oxford, UK), the library was then sequenced on the PromethION (Oxford Nanopore Technologies; Wang et al. 2021).

### 5.2 Identification of Polyploidy, Estimation of Genome Size and Heterozygosity

A *k*‐mer analysis was performed using MGI sequencing data prior to genome assembly to estimate genome size and heterozygosity with KMC2 3.2.1 (Deorowicz et al. 2015). By analysing the 17‐mer depth distribution, the genome size was estimated, and by further combining the simulation data results of Arabidopsis with different heterozygosity and the frequency peak distribution of 17 *k*‐mer, the heterozygosity and repeat content of *A. valvata* genome were estimated.

### 5.3 Genome Assembly and Assessment

A draft genome was assembled with hifiasm software (Cheng et al. 2021) using the HiFi data. To obtain uniquely mapped paired‐end reads, the clean HiC paired‐end reads were mapped to the draft sequence assembly with bowtie2 (v2.3.2) (Langmead and Salzberg 2012). The alignment parameters were set to ‐end‐to‐end and ‐very‐sensitive with an ‐L value of 30. HiC‐Pro (v2.8.1) (Servant et al. 2015) was used to identify and retain the valid interacting paired reads from the uniquely mapped paired‐end reads for analysis. The scaffolds were clustered through hierarchical clustering by LACHESIS (Burton et al. 2013), with parameters cluster_min_re_sites = 100, cluster_max_link_density = 2.5, cluster_noninformative_ratio = 1.4, order_min_n_res_in_trunk = 60. Then, scaffolds were ordered and oriented based on the genome sequence of the *A. chinensis* cultivar ‘Hongyang’ v3 (Wu et al. 2019). Python software was used to create a Hi‐C interaction heatmap of pseudochromosomes to evaluate the effectiveness of genome assembly.

A thorough quality assessment was conducted using assembly QC (Rashid et al. 2024), which includes multiple QC tools such as FCS tools (FCS‐adaptor and FCS‐gx) (Sayers et al. 2023) for adapter and contaminants detection, BUSCO (Simão et al. 2015) for gene space completeness, Kraken2 for contamination check and taxonomy confirmation, and quarTeT was used to identify centromeres and telomeres (Lin et al. 2023). Moreover, NGS and HiFi reads were mapped back to the assembly using bowtie2 (v2.3.2) to check the coverage and sequencing depth across the chromosomes; subgenome phasing was performed using SubPhaser (Jia et al. 2022), CRAQ (Li et al. 2023) and Merqury (Rhie et al. 2020) were used to perform genome quality checking.

### 5.4 Repeat Annotation and Gene Prediction

Tandem repeats were annotated using GMATA v2.2 (Wang and Wang 2016) for simple sequence repeats (SSRs) and Tandem Repeats Finder (TRF) v 4.07b (Benson 1999) for all tandem repeat elements across the genome. Transposable elements (TEs) were then identified using a combination of ab initio and homology‐based methods. Three independent approaches were used for gene prediction, namely ab initio prediction, homology search and reference guided transcriptome assembly. Gene function information, motifs and domains of their encoding proteins were assigned by comparing sequences with the public databases SwissProt, NCBI NR, KEGG, KOG and GO. The putative domains and GO terms of genes were identified using InterProScan with default parameters (Zdobnov and Apweiler 2001). For the other four databases, BLASTp was used to compare the protein sequences obtained from Evidence Modeller‐integrated (EVM) (Haas et al. 2008) against the four public protein databases with an E value cutoff of 1e^−05^; the hit with the lowest E value was retained. Results from the five database searches were concatenated. To identify non‐coding RNAs (ncRNAs), two strategies were used: searching against databases and prediction with models. Transfer RNAs (tRNAs) were predicted using tRNAscan‐SE v2.0 with eukaryotic parameters (Lowe and Eddy 1997). MicroRNAs, ribosomal RNA (rRNA) and small nuclear RNAs were detected using Infernal to search the Rfam database (Griffiths‐Jones et al. 2005; Nawrocki and Eddy 2013). The rRNAs and their encoded subunits were predicted using RNAmmer v1.2 (Lagesen et al. 2007).

### 5.5 Homeologs‐Aware Gene Annotation

Homeologs‐aware set was defined by identifying homologous genes at the same locus using AlleleFinder (https://github.com/sc‐zhang/AlleleFinder), using the *A. chinensis* ‘Hongyang’ v3 genome as a reference.

### 5.6 Genome Comparison and Synteny Analysis

Minimap v2.26 (‐ax asm10) was used to compare the six haplotype‐resolved assemblies to the ‘Hongyang’ v3 genome. A collinearity map was plotted using the NGenomeSyn tool (Li 2018). Pairwise comparisons between the six haplotype assemblies were conducted using minimap v2.26 (‐ax asm10); syri v1.6.3 was used to draw the syntenic blocks (Goel et al. 2019). The public genome data were downloaded from the KPGD database (Li, Li, et al. 2025).

### 5.7 Reconstruction of Phylogenetic Trees and Analysis of Gene Families

OrthoMCL (Li et al. 2003) was used to identify homologous and orthologous gene sets among *A. valvata* and 11 other taxa: *Oryza sativa* (Sakai et al. 2013), *Arabidopsis thaliana* (Swarbreck et al. 2008), *Vitis vinifera* (Velt et al. 2023), *Rhododendron delavayi* (Zhang et al. 2017), *A. chinensis* ‘Hongyang’ (Wu et al. 2019), *A. eriantha* ‘White’ (Tang et al. 2019), *A. arguta* ‘Longcheng No 2’ (Zhang et al. 2024), *A. chinensis* . var. *A. deliciosa* (Liu et al. 2024), *A. hemsleyana* (Yu et al. 2023), *A. macrosperma* (Li et al. 2024), and *A. polygama* (Li et al. 2024). Based on the orthologous gene sets identified by OrthMCL, a phylogenetic analysis was performed using the shared single‐copy orthologous genes. Based on the resulting phylogenetic tree, the RelTime method implemented in MEGA‐CC (Kumar et al. 2012) was used to compute the mean substitution rates along each branch and estimate the species divergence times. Three fossil calibration times were obtained from the TimeTree database (http://www.timetree.org/) as time controls, namely the divergence times of Arabidopsis (148–173 million years ago [Mya]), and rice (40–53 Mya). OrthoFinder (Emms and Kelly 2019) was used to identify orthologous gene sets, infer gene trees and species tree among *A. valvata* and seven *Actinicia* species.

Detection of gene family expansion and contraction, which are often associated with adaptive divergence of closely related species, was performed using CAFE (De Bie et al. 2006). Average *Ka*/*Ks* values were calculated before conducting a branch‐site likelihood ratio test using Codeml implemented in the PAML package (Yang 2007) to identify positively selected genes among *A. valvata* and the 11 other plant taxa. Genes with a *p*‐value < 0.05 under the branch‐site model were considered positively selected genes (Yang 2007). Synonymous substitution rate (*Ks*) estimation was used to detect WGD events in the *A. valvata* genome and other species.

### 5.8 Subcellular Localisation and Transactivation Activity Assay in Yeast

For subcellular localisation of the protein encoded by 16B1g14180, its full‐length coding sequence was inserted into the pHB‐YFP vector after BamHI/SpeI digestion. Protoplasts prepared from 4‐week‐old rosette leaves of Arabidopsis plants were transfected with *YFP* or 16B1g14180‐*YFP* plasmids using the polyethylene glycol (PEG) method. YFP fluorescence was visualised 18 h post‐transfection with an Olympus FV1000 confocal microscope (Tokyo, Japan).

The transactivation activity of the protein encoded by 16B1g14180 was assayed in yeast. Full‐length or truncated fragments of 16B1g14180 were cloned in‐frame with the sequence encoding the DNA‐binding domain of yeast GAL4 in the pGBKT7 vector; the resulting plasmids were introduced separately into yeast strain AH109. The growth of positive transformants was evaluated by serial dilution on synthetic defined (SD)/−Trp/−His/−Ade medium containing X‐α‐Gal.

### 5.9 Kiwifruit Transgene and *ERF* Gene Function Identification in Waterlogging

The full‐length coding sequence of 16B1g14180 was cloned into the pBI121 vector using the XbaI and BamHI restriction sites. The resulting *35S*:16B1g14180 plasmid was introduced into Agrobacterium ( *Agrobacterium tumefaciens* ) strain EHA105. Transformation of *A. chinensis* ‘Hongyang’ was carried out following a previously published protocol. Leaf strips were transferred to co‐cultivation medium, and emerging adventitious buds were moved to shoot‐induction medium containing 100 mg L^−1^ kanamycin for incubation for 30 days. Surviving shoots were transferred to rooting medium, and clonal transgenic plants were maintained in a greenhouse. Newly emerging leaves from the transgenic plants were collected for DNA extraction, and the presence of the 35S and 16B1g14180 transgenic sequences was verified using primers 35S:16B1g14180‐F/R.

Transgenic plants from two independent lines (OE 1 and OE 2) and control ‘Hongyang’ plants were grown in a greenhouse at 25°C–28°C. Healthy, 50‐day‐old plants in 12.5‐cm‐diameter pots were selected for waterlogging treatment. Six pots (two per genotype) were placed in a plastic container (45 cm × 35 cm × 16 cm) filled with water maintained 2–3 cm above the soil surface. After 12 h of waterlogging, roots were harvested from six plants (three replicates of two plants each) pooled per genotype for measurements of sugar contents. Glucose and fructose contents were measured by high‐performance liquid chromatography according to a previous procedure. After 14 days of waterlogging, the death rate and aboveground fresh and dry weights were recorded (three replicates of six plants each per genotype). Dry weight was measured after drying at 65°C for 2–3 days.

## Author Contributions

M.L. and X.Q. designed the research. M.L., Z.L., C.H.D., C.L., Z.Z., Q.Z., Y.L., P.Z., L.S., M.L., J.F. conducted the experiments and analysed the data, M.L., Z.L., C.H.D. and X.Q. wrote the manuscript.

## Funding

This work was supported by the China Agriculture Research System of MOF and MARA (CARS‐26), the National Key Research and Development Program of China (2022Y1600701) and the Special Funds for Science and Technology Innovation Project of the Chinese Academy of Agricultural Sciences (CAAS‐ASTIP‐2024‐ZFRI).

## Conflicts of Interest

The authors declare no conflicts of interest.

## Supporting information


**Figure S1:** Genome survey of *A. valvata* ZK2.
**Figure S2:** Quality, numbers and average GC content of MGI sequencing short reads.
**Figure S3:** Comparative collinearity and structural variation analysis between A1 and the two putative progenitors of *A. valvata*.
**Figure S4:** Comparative collinearity and structural variation analysis between B1 and the two putative progenitors of *A. valvata* .
**Figure S5:** Hi‐C interactions across the *A. valvata* genome assembly. Strong interactions are indicated in dark red.
**Figure S6:** CRAQ plot of the six haplotype genomes.
**Figure S7:** Phased subgenomes of hexaploid *A. valvata* genome.
**Figure S8:**. Telomere analysis of haplotype‐resolved genome of A1 genome.
**Figure S9:** The sequencing depth and coverage analyses of HiFi and NGS data mapping to *A. valvata* ZK2 haplotype A1 genome.
**Figure S10:** Switch error evaluation by ultra‐long ONT.
**Figure S11:**. Sequence divergence rate of TEs in the six haplotypes.
**Figure S12:** Phylogenetic tree inferred using OrthoFinder.
**Figure S13:** GO term and KEGG pathway analysis of expanding gene families in the haplotype B genome assembly.
**Figure S14:** Sequence alignment of the one homoeologous gene from *ERF* genes across the six haplotypes.

**Table S1:** MGI sequencing data of *A. valvata* ‘Zhongmikangzhen No 2’.
**Table S2:** PacBio Revio sequencing data of *A. valvata* ‘Zhongmikangzhen No 2’.
**Table S3:** Hi‐C sequencing data of *A. valvata* ‘Zhongmikangzhen No 2’.
**Table S4:** Summary of the number and size of contigs in each haplotype‐resolved genome assembly.
**Table S5:** Merqury quality assessment metrics across the six haplotypes.
**Table S6:** Statistical analysis of telomere distribution across all chromosomes in the haplotype‐resolved genome assembly.
**Table S7:** Identification of centromeres in each haplotype‐resolved genome assembly.
**Table S8:** Statistics of sequencign depth for HiFi and NGS data.
**Table S9:** Summary of annotated transposable elements and repetitive sequences in the six haplotype‐resolved assemblies.
**Table S10:** Summary of annotated genes using the five databases for the six haplotype‐resolved assemblies.
**Table S11:**. Identification of tRNA, rRNA and ncRNA in the six haplotype‐resolved genomes assemblies.
**Table S12:**. BUSCO analysis of annotated genes.
**Table S13:** The genes with homoeolog‐aware genes in the six haplotype genomes.
**Table S14:**. Variation analysis of SNP, InDels, translocation, duplication and inversion in six haplotype genomes.
**Table S15:** Significant GO terms and KEGG pathways enriched in expanded genes.
**Table S16:**. The gene expression of *ERF* II gene family under waterlogging stress.
**Table S17:** Primers used in this study.

## Data Availability

Raw PacBio, Hi‐C sequencing read, ONT data and transcriptome reads and assemblies for the *A. valvata* genomes have been deposited in CNGB Sequence Archive (CNSA) of China National GeneBank DataBase (CNGBdb) under the accession number PRJCA053631. The *ERF* gene sequence has been submitted to NCBI genbank (Genbank No: PZ229133).
