# Supplementary material for: High‐Contiguity Haplotype‐Resolved Genome Assembly of the Hexaploid Actinidia valvata Rootstock Sheds Light on Waterlogging Resistance Gene
Source: Plant Biotechnol J. 2026 May 29:10.1111/pbi.70695. Online ahead of print. doi: 10.1111/pbi.70695 (PMC13398721; doi:10.1111/pbi.70695)
Supplement: Supplementary file 1 — Figure S1: Genome survey of A. valvata ZK2. Figure S2: Quality, numbers and average GC content of MGI sequencing short reads. Figure S3: Comparative collinearity and structural variation analysis between A1 and the two putative progenitors of A. valvata. Figure S4: Comparative collinearity and structural variation analysis between B1 and the two putative progenitors of A. valvata . Figure S5: Hi‐C interactions across the A. valvata genome assembly. Strong interactions are indicated in dark red. Figure S6: CRAQ plot of the six haplotype genomes. Figure S7: Phased subgenomes of hexaploid A. valvata genome. Figure S8:. Telomere analysis of haplotype‐resolved genome of A1 genome. Figure S9: The sequencing depth and coverage analyses of HiFi and NGS data mapping to A. valvata ZK2 haplotype A1 genome. Figure S10: Switch error evaluation by ultra‐long ONT. Figure S11:. Sequence divergence rate of TEs in the six haplotypes. Figure S12: Phylogenetic tree inferred using OrthoFinder. Figure S13: GO term and KEGG pathway analysis of expanding gene families in the haplotype B genome assembly. Figure S14: Sequence alignment of the one homoeologous gene from ERF genes across the six haplotypes. [file PBI-9999-0-s001.docx]

**
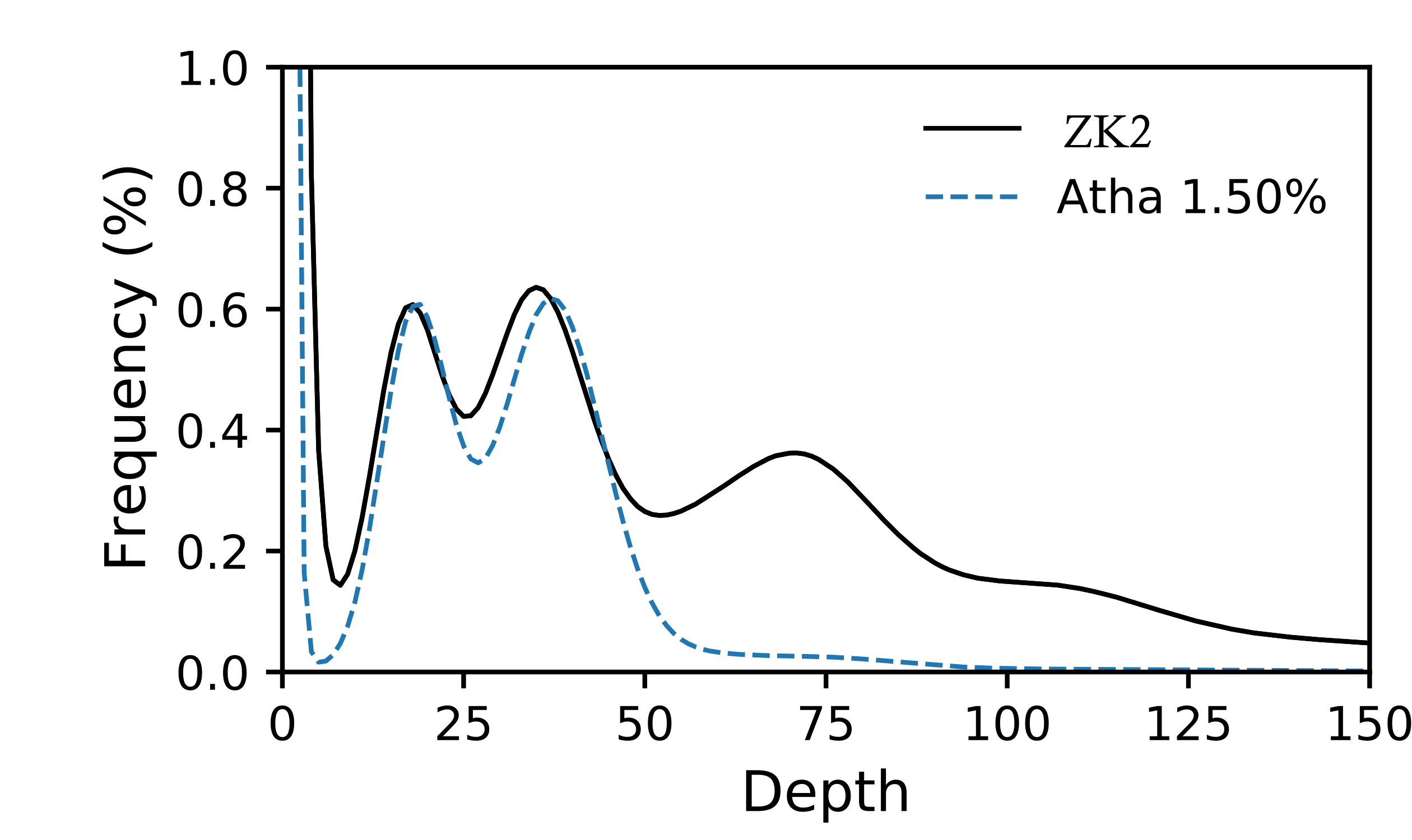
**
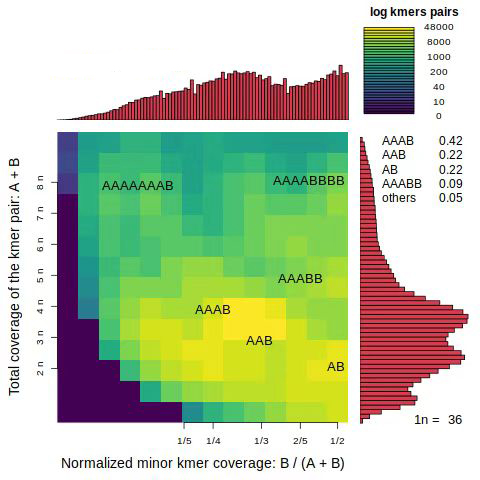


B

A

**Figure S1.** Genome survey of *A. valvata* ZK2. **A** *k*-mer distribution and heterozygosity simulation curve. **B** Smudgeplot analysis of *k*-mer pairs.


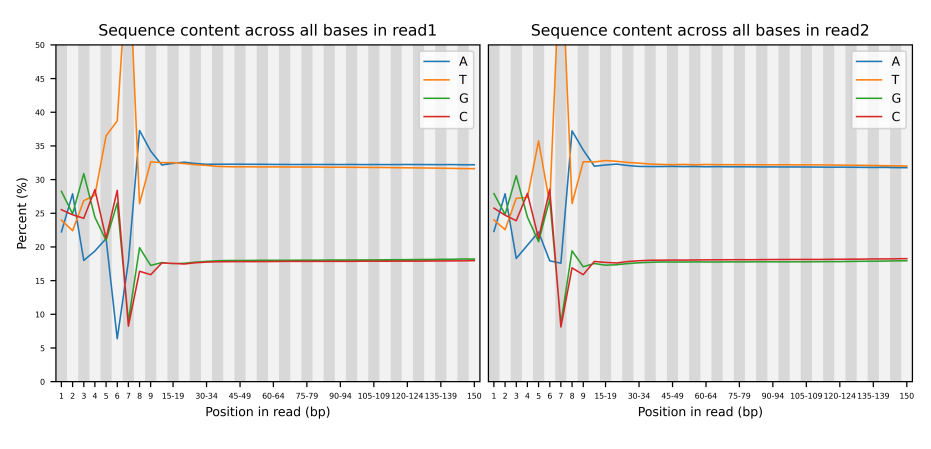


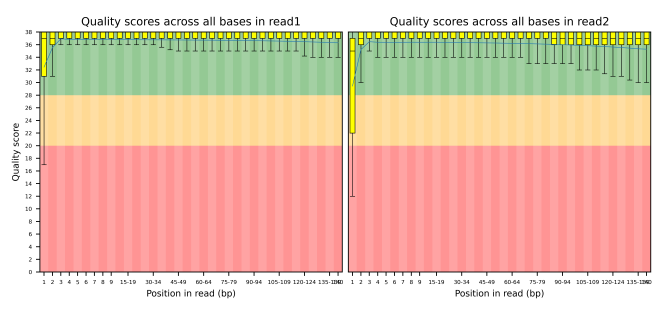


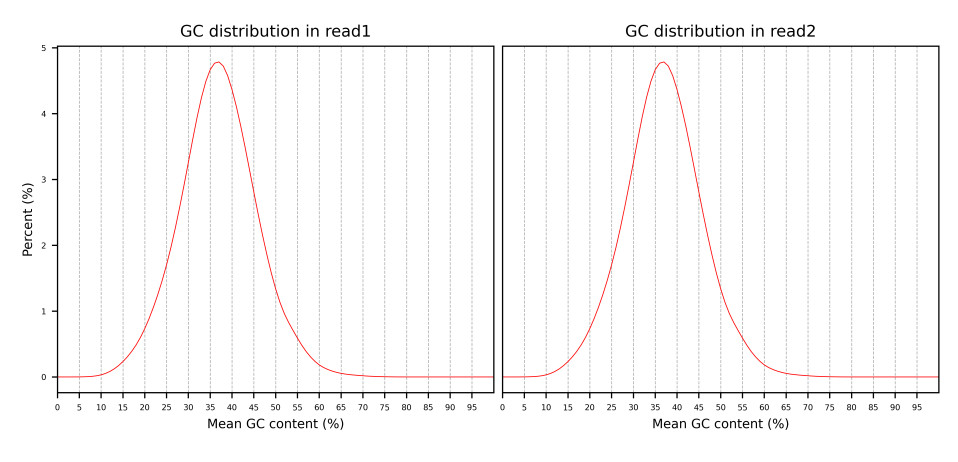


**Figure S2.** Quality, numbers, and average GC content of MGI sequencing short reads.
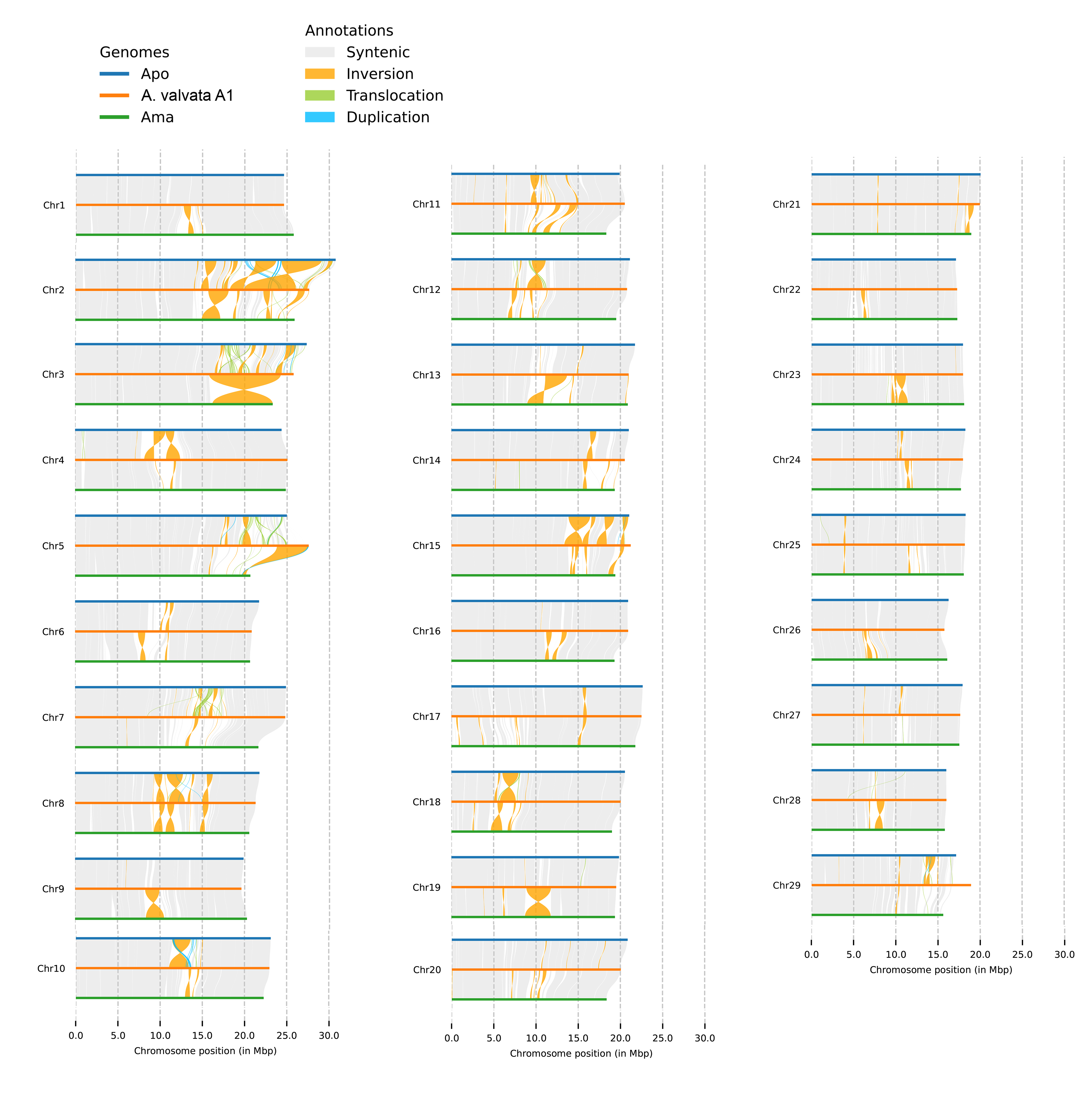


**Figure S3.** Comparative collinearity and structural variation analysis between A1 and the two putative ancestors of hexaploid *A. valvata*. Apo is *polygama*, Ama is *A. macrosperma.*


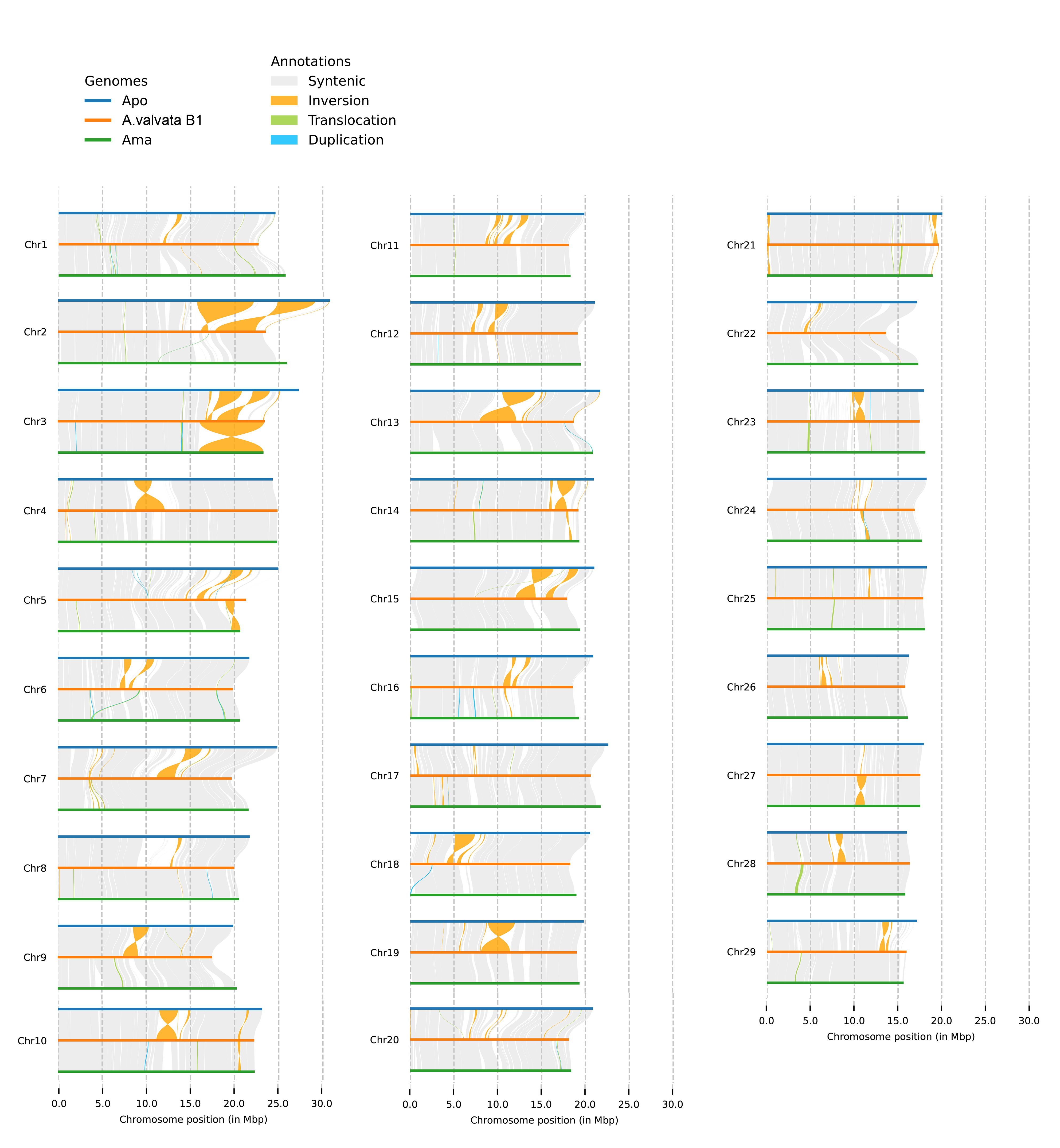


**Figure S4.** Comparative collinearity and structural variation analysis between B1 and the two putative ancestors of hexaploid *A. valvata*. Apo is *polygama*, Ama is *A. macrosperma.*

**
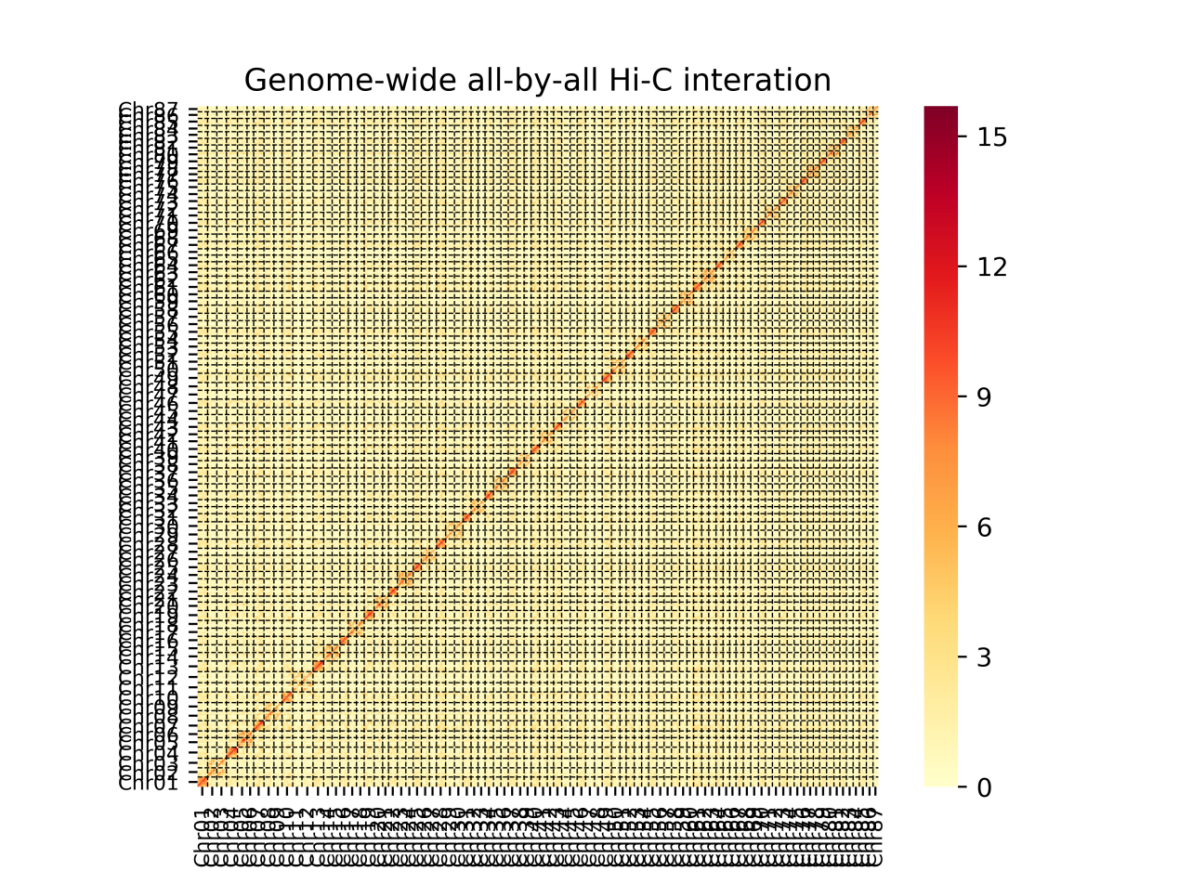
**

**Figure S5.** Hi-C interactions of *A. valvata* ZK2*.* Strong interactions are indicated in dark red


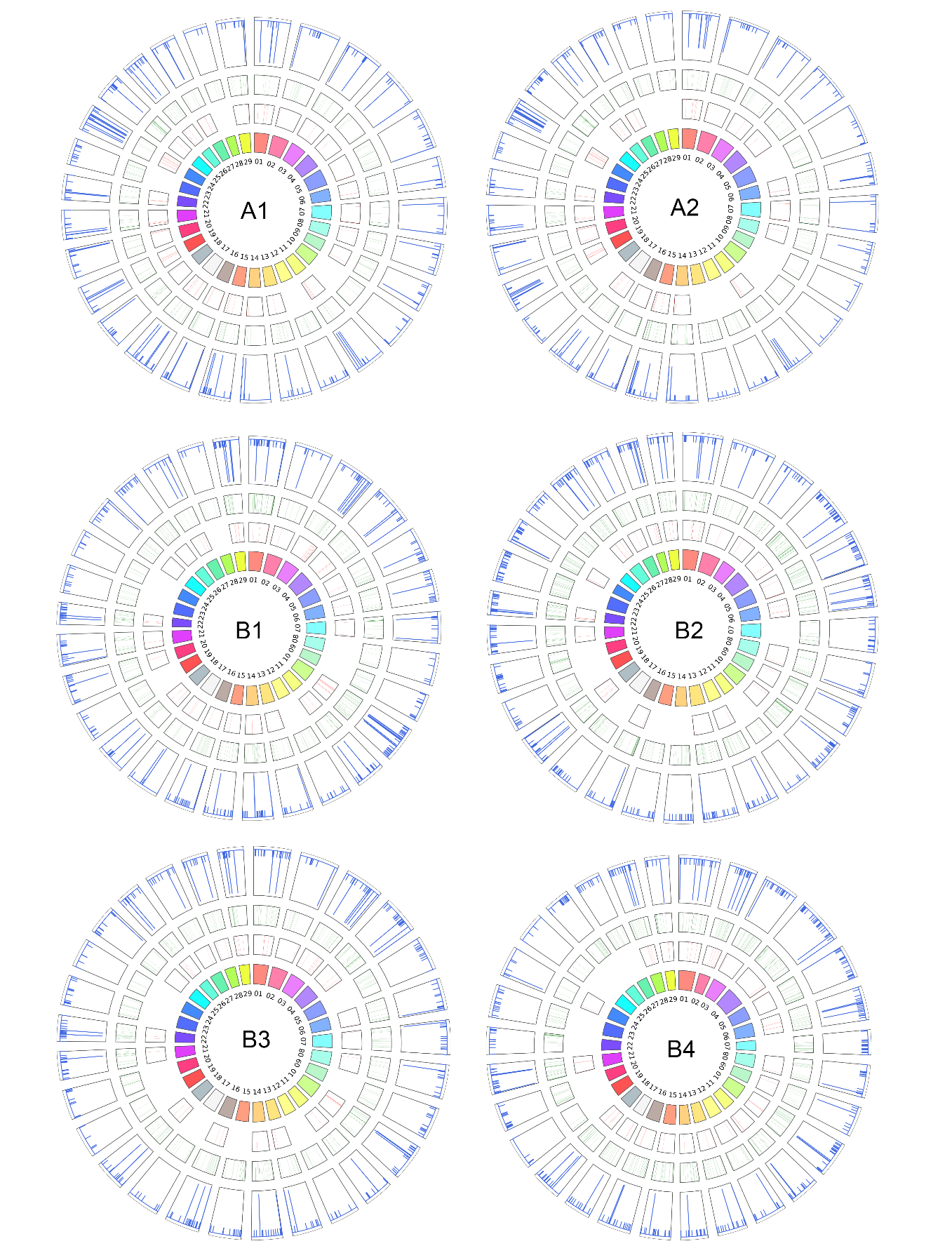


**Figure S6.** CRAQ plot of the six haplotype genomes.


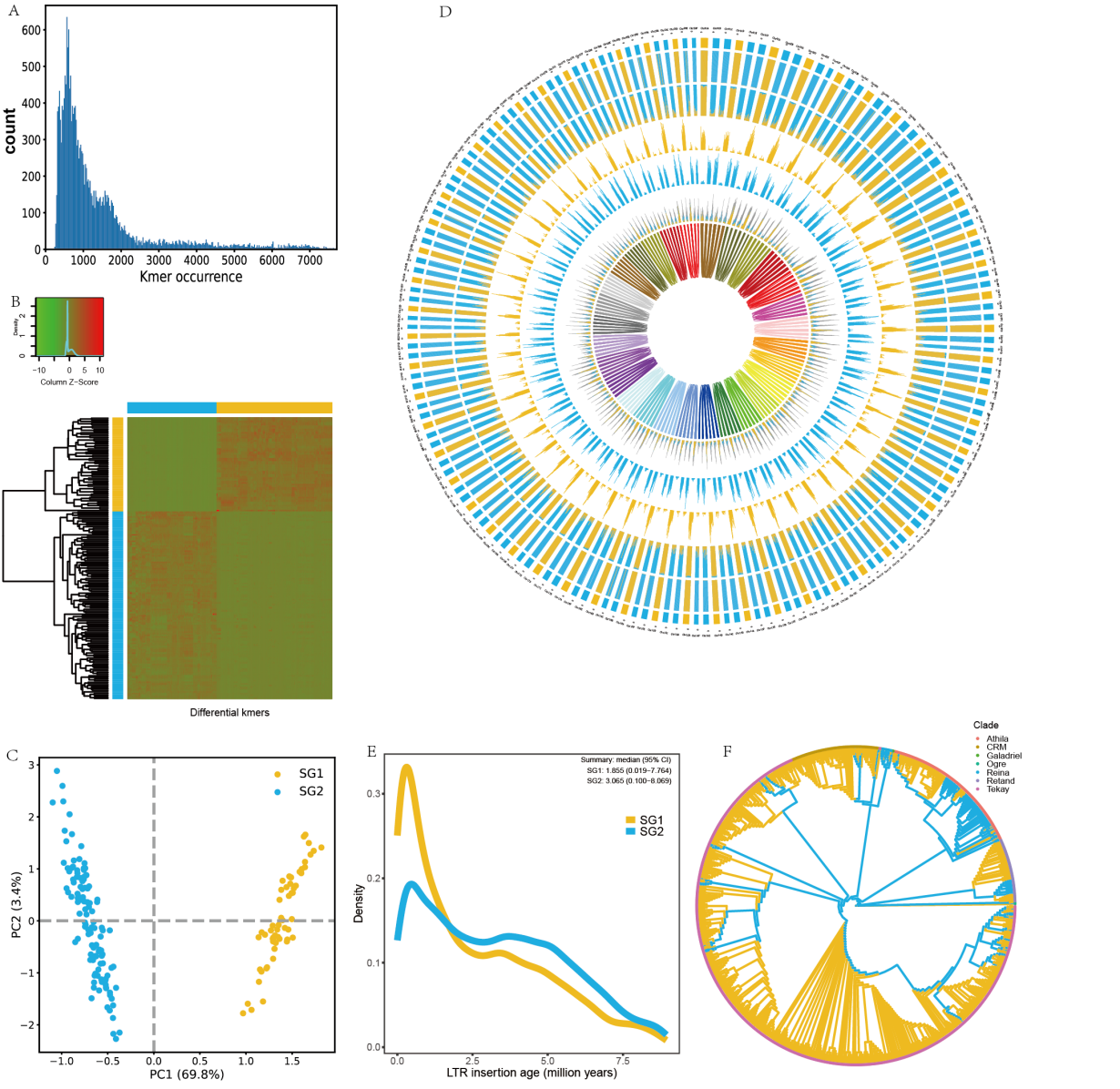


**Figure S7.** Phased subgenomes of hexaploid *A.valvata* genome. Colors are unified with each subgenome in subplots B-F, i.e. the same color means the same subgenome. SG1: A1 and A2; SG2: B1, B2, B3, B4.

**A** The histogram of differential *k*-mers among homoeologous chromosome sets. **B** Heatmap and clustering of differential *k*-mers. **C** Principal component analysis (PCA) of differential *k-*mers. Points indicate chromosomes. **D** Chromosomal characteristics (window size: 1 Mb). **E** Insertion time of subgenome-specific LTR-RTs. **F** A phylogenetic tree of 1,000 randomly subsampled LTR/Gypsy elements.


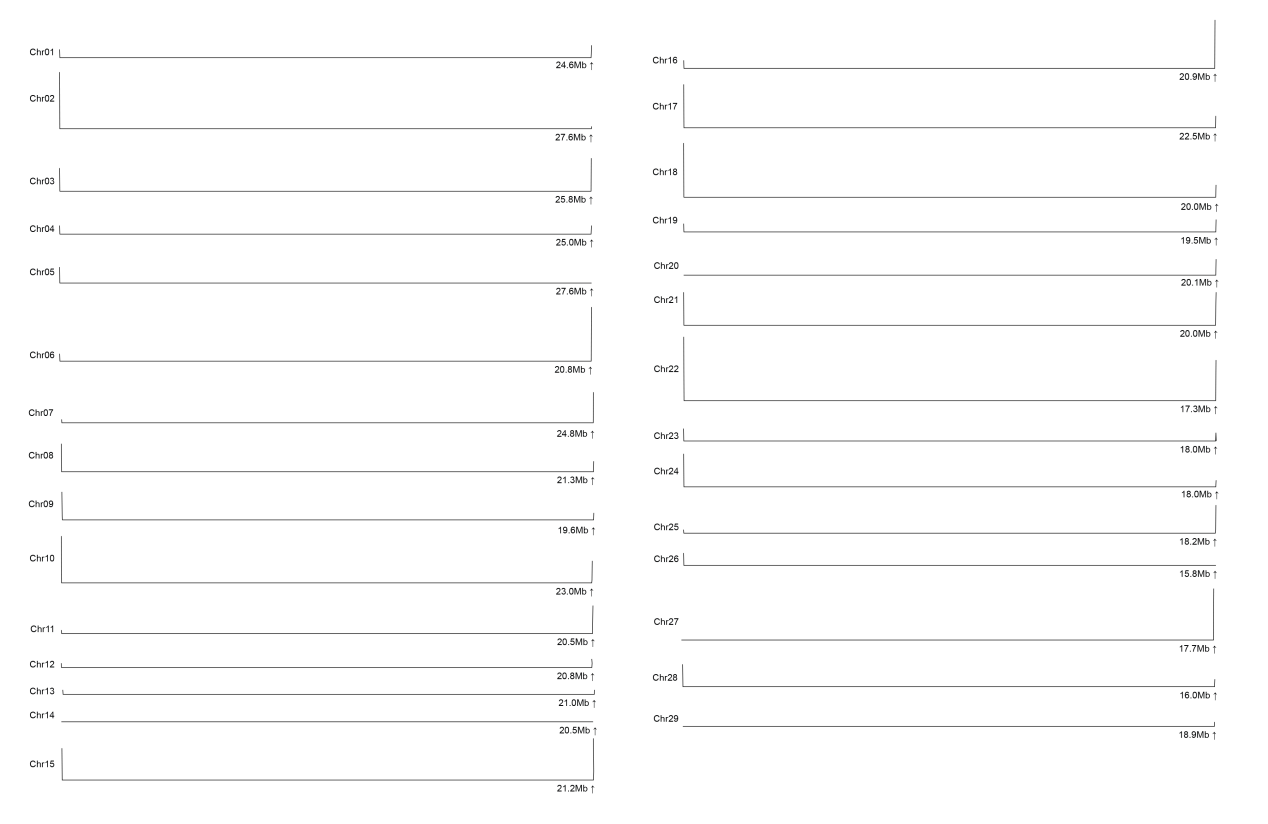


**Figure S8.** Telomere analysis of haplotype-resolved genome of A1 genome.


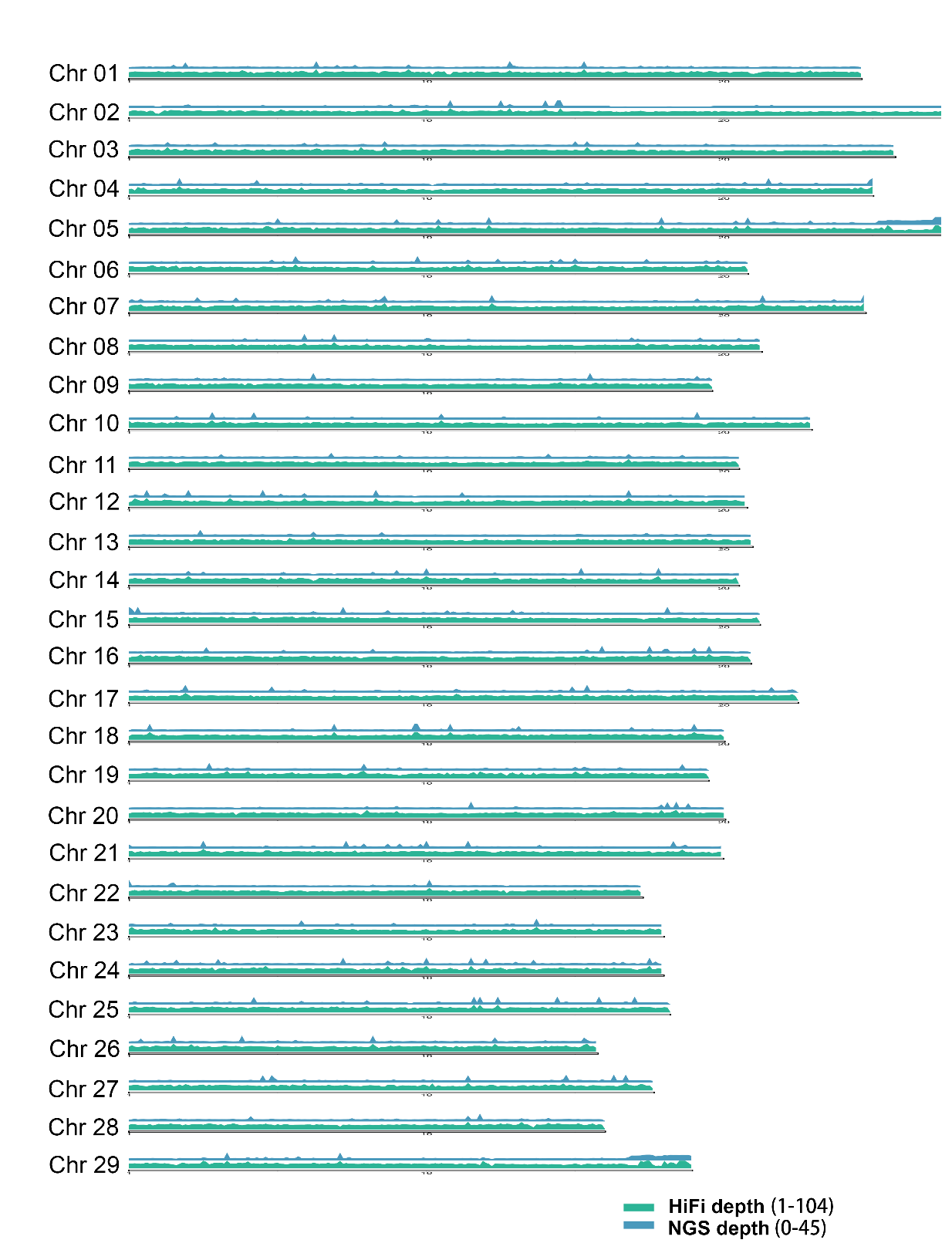


**Figure S9.** The depth coverage of HiFi and NGS aligned with *A. valvata* ZK2 haplotype A1 genome.

**
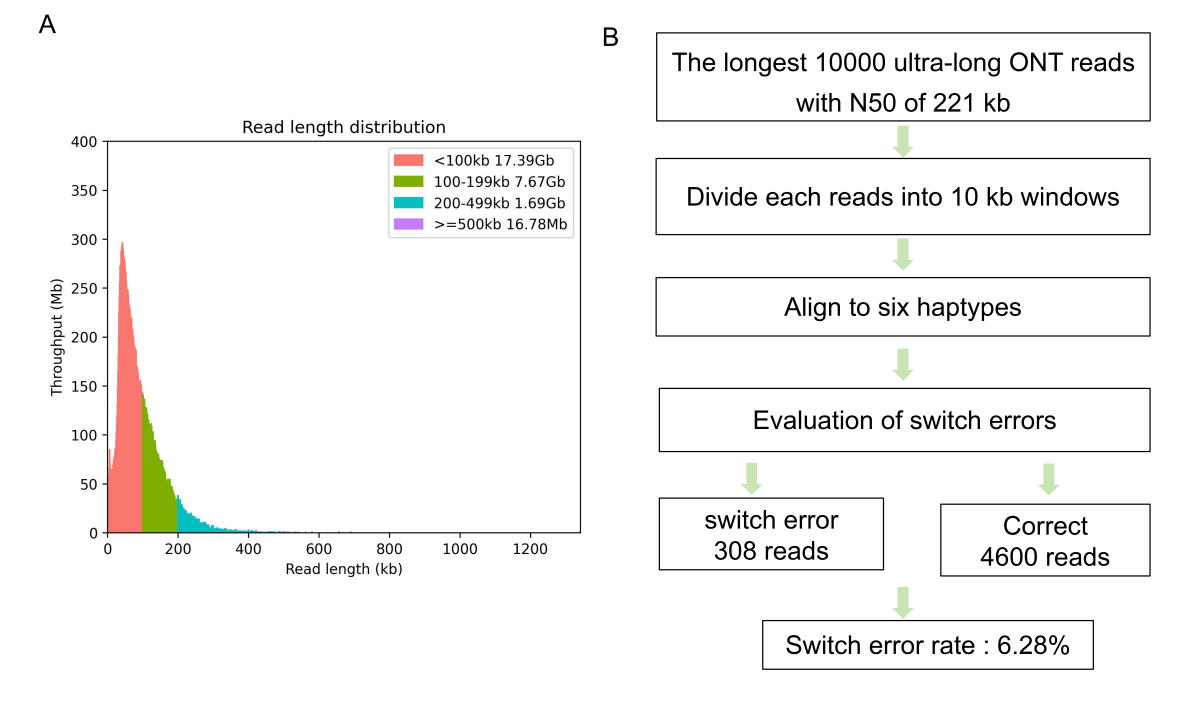
**

**Figure S10.** Switch error evaluation by ultra-long ONT. A total of 18.7 G ONT reads were generated and used to evaluated the switch error.

**A** Length distribution by ONT Ultra-long sequencing. **B** Schematic diagram of switch errors evaluation. The ONT data was corrected with Illumina reads, and the longest 10000 ultra-long ONT reads with N50 of 221 kb were aligned against each 10 kb windows to six haplotypes of *A. valvata.*


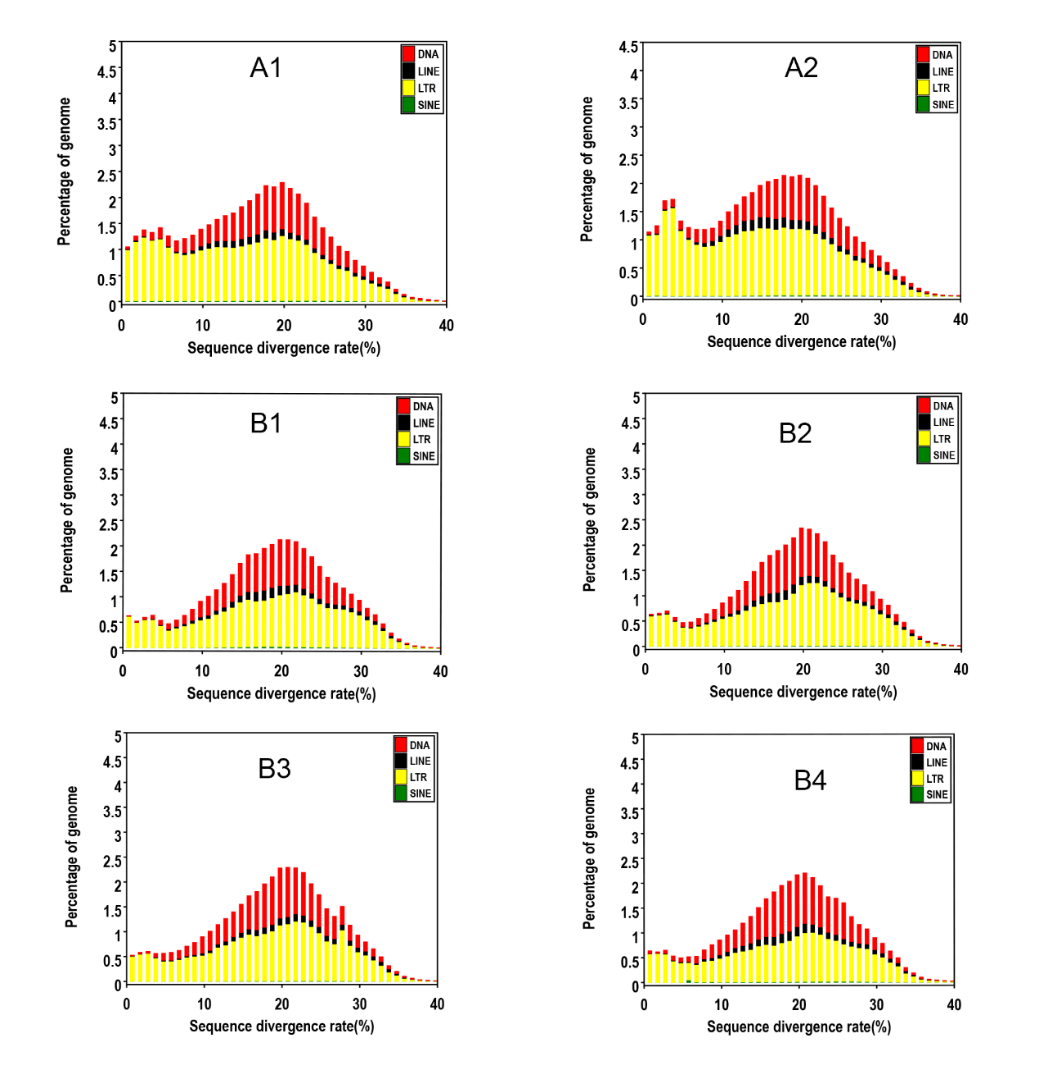


**Figure S11.** Sequence divergence rate of TEs in the six haplotypes.


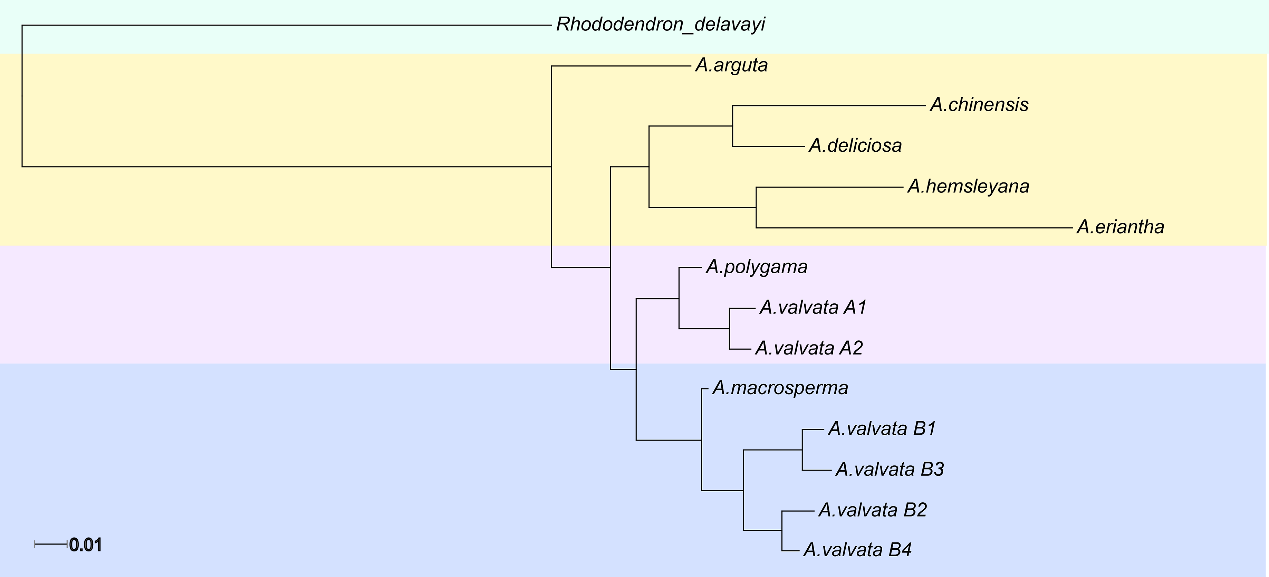


**Figure S12.** Phylogenetic tree inferred using OrthoFinder.

**
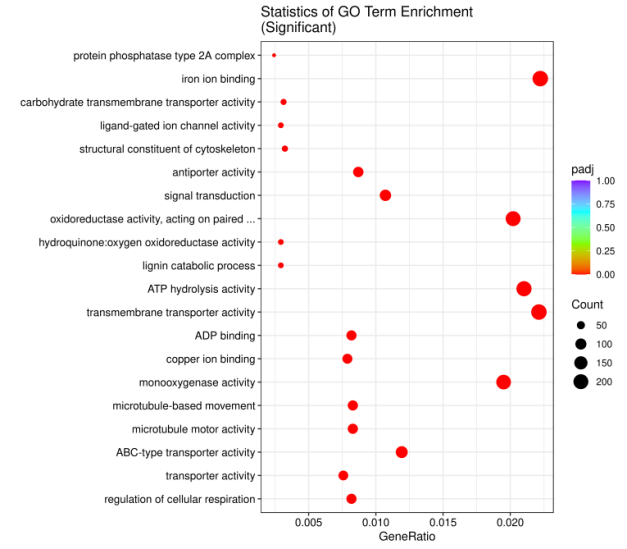

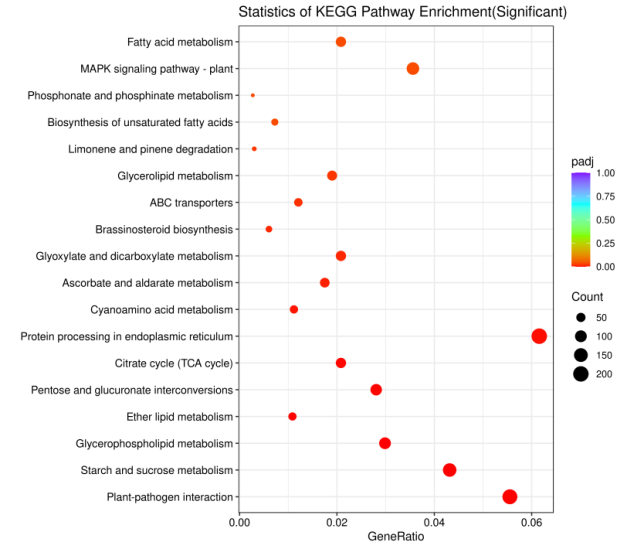
**

**Figure S13.** GO term and KEGG pathway analysis of expanding gene families in the haplotype B genome assembly.


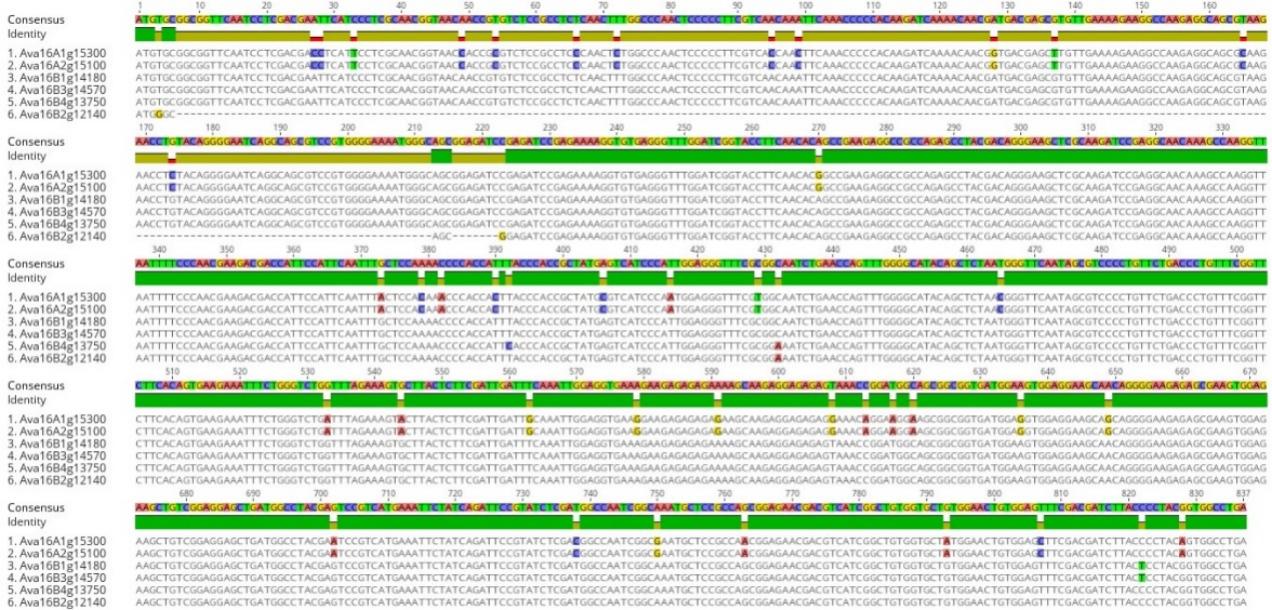


**Figure S14.** Sequence alignment of the one homoeologous gene from *ERF* genes across the six haplotypes.
